# Integrated Approaches in Drug Repositioning Highlight Ouabain and Helenalin as Potential Drug Candidates for Pulmonary Fibrosis

**DOI:** 10.34133/csbj.0193

**Published:** 2026-08-10

**Authors:** Zeyad Al-Abdulraheem, Hanna-Kaarina Juppi, Jack Morikka, Simo Inkala, Noora Perho, Saara Sani, Angela Serra, Antonio Federico, Michele Fratello, Dario Greco

**Affiliations:** ^1^ Finnish Hub for Development and Validation of Integrated Approaches (FHAIVE), Faculty of Medicine and Health Technology, Tampere University, 33100 Tampere, Finland.; ^2^ Tampere Institute for Advanced Study, Tampere University, Tampere, Finland.; ^3^ Division of Pharmaceutical Biosciences, Faculty of Pharmacy, University of Helsinki, 00100 Helsinki, Finland.

## Abstract

New approaches to drug discovery are urgently needed, especially for diseases with complex molecular mechanisms where current treatments show limited efficacy. We present a data-driven workflow for drug repositioning that integrates high-throughput and virtual screening, molecular docking, and mechanistic pharmacokinetic modeling with biological validation using lung fibrosis as a model disease. High-throughput screening assays and LINCS L1000, SwissTarget, and Unified Knowledge Space (UKS) databases identified ouabain and helenalin as candidates targeting lung fibrosis-related genes. Pharmacophore modeling, docking analysis, and physiologically based pharmacokinetic modeling confirmed drug-like properties comparable to current lung fibrosis treatments. In silico findings were validated using RNA-sequencing data from idiopathic pulmonary fibrosis patients and quantitative polymerase chain reaction data from human alveolar epithelial cells exposed to profibrotic transforming growth factor-β. In vitro, helenalin and ouabain induced distinct gene expression profiles. The anti-inflammatory signature observed for helenalin points to preliminary functional potential warranting further investigation. This workflow can be generalized across diverse therapeutic areas to accelerate cost-effective drug discovery and repositioning.

## Introduction

The design and development of novel drugs are both expensive and time-consuming endeavors, often spanning over a decade and costing billions of U.S. dollars [1]. Consequently, repurposing existing drugs for pathologies distinct from their original therapeutic indication has emerged as a favorable strategy, due to reduced development costs and expedited timelines. For many years, target-based high-throughput screening (HTS) has been a cornerstone in drug discovery, leading to the identification of numerous molecules that can modulate or inhibit targets relevant to specific pathologies. Although widely employed, HTS alone is limited by its inability to capture complex [2–4] biological interactions and requires secondary validation with physiologically relevant models. HTS also provides no indication of the pharmacokinetics and pharmacodynamics of screened compounds [5]. Addressing these limitations necessitates a combination of data-driven drug discovery, computer-aided drug design, and in-depth analysis of the biological processes affected by the disease and drug activity. Yet, current drug discovery approaches are often limited by fragmented data integration and a focus on isolated pathways over the complex interplay of molecular networks [6]. Traditional approaches also neglect linking chemical structural features to pharmacological efficacy [7], and reviews of computational methods highlight the persistent disconnect between in silico predictions and experimental validation, further stressing the need for integrated approaches in drug discovery pipelines [8–12].

Among conditions with marked unmet medical needs, lung diseases represent a notable healthcare burden, encompassing fibrosis types arising from lung inflammation or injury leading to progressive scar tissue formation [13]. Idiopathic pulmonary fibrosis (IPF), chronic obstructive pulmonary disease (COPD), and cystic fibrosis (CF) are examples of pathologies showing high morbidity [14]. Central to the fibrotic process is transforming growth factor-β (TGF-β), which, upon release by lung cells, drives fibroblast differentiation into myofibroblasts, leading to matrix stiffening [15]. Repeated injury of alveolar epithelium is widely considered a key initiating event in pulmonary fibrosis, triggering the release of TGF-β and other profibrotic signals that amplify this cascade [16]. Other key contributors to lung inflammation include interleukin-1β (IL-1β), tumor necrosis factor-α (TNF-α), and nuclear factor κB (NF-κB) signaling [17,18]. Separate from other fibrosis types, CF results from *CFTR* gene mutations, but CFTR dysfunction is also implicated in IPF and COPD [19–22]. IPF, COPD, and CF are progressive pathologies currently lacking definitive treatment. U.S. Food and Drug Administration (FDA)-approved therapies include nintedanib, pirfenidone, nerandomilast, ivacaftor, and dupilumab [2–4,23], which have demonstrated the ability to decelerate disease progression, but are associated with a range of side and adverse effects, such as upper respiratory tract infection, and gastrointestinal, neurological, and dermatological issues [24,25]. Additionally, their capacity for substantial disease improvement remains limited [24,25]. Thus, discovering more effective therapeutics for lung fibrosis is desirable, and a drug repositioning via in silico exploration is warranted.

To address this gap, our study adopted an integrative approach to drug discovery, using lung fibrosis as a case study. By using publicly available databases, in-house knowledge graph called the Unified Knowledge Space (UKS) [ 26–29], computational data analysis, and biological validation, we identified 2 interesting drug candidates, ouabain and helenalin, capable of targeting multiple molecular endpoints in lung fibrosis. The approach used is generalizable beyond lung fibrosis, holding promise for therapeutic discovery in other complex diseases, potentially accelerating the broader field of drug discovery.

## Results

### Data-driven approaches reveal ouabain and helenalin as potential drug candidates for lung fibrosis

**Fig. 1.**
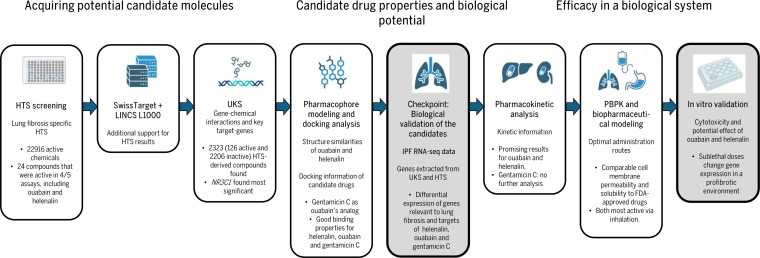
Workflow of the drug candidate discovery for lung fibrosis.

To identify drug targets for lung fibrosis, we analyzed data from 5 lung fibrosis-related HTS bioassays annotated in the National Center for Biotechnology Information (NCBI) PubChem database [30]. The bioassays, screening for CFTR agonists, NF-κB inhibitors, TNF-α -inhibitors, IL-1β inhibitors, and TGF-β inhibitors, were selected based on their contribution to lung fibrosis and role as FDA-approved treatment targets [2–4]. Altogether, the HTS bioassays contained 1,697,299 bioactivity tests quantifying 509,527 unique compounds (Fig. 1). Of these, 22,916 compounds were classified as active in at least one bioassay. From the active compounds, 24 were shared among at least 4 of 5 bioassays (CFTR, NF-κB, IL-1β, and TGF-β). The full list of the shared compounds is presented in Table S1.

**Fig. 2.**
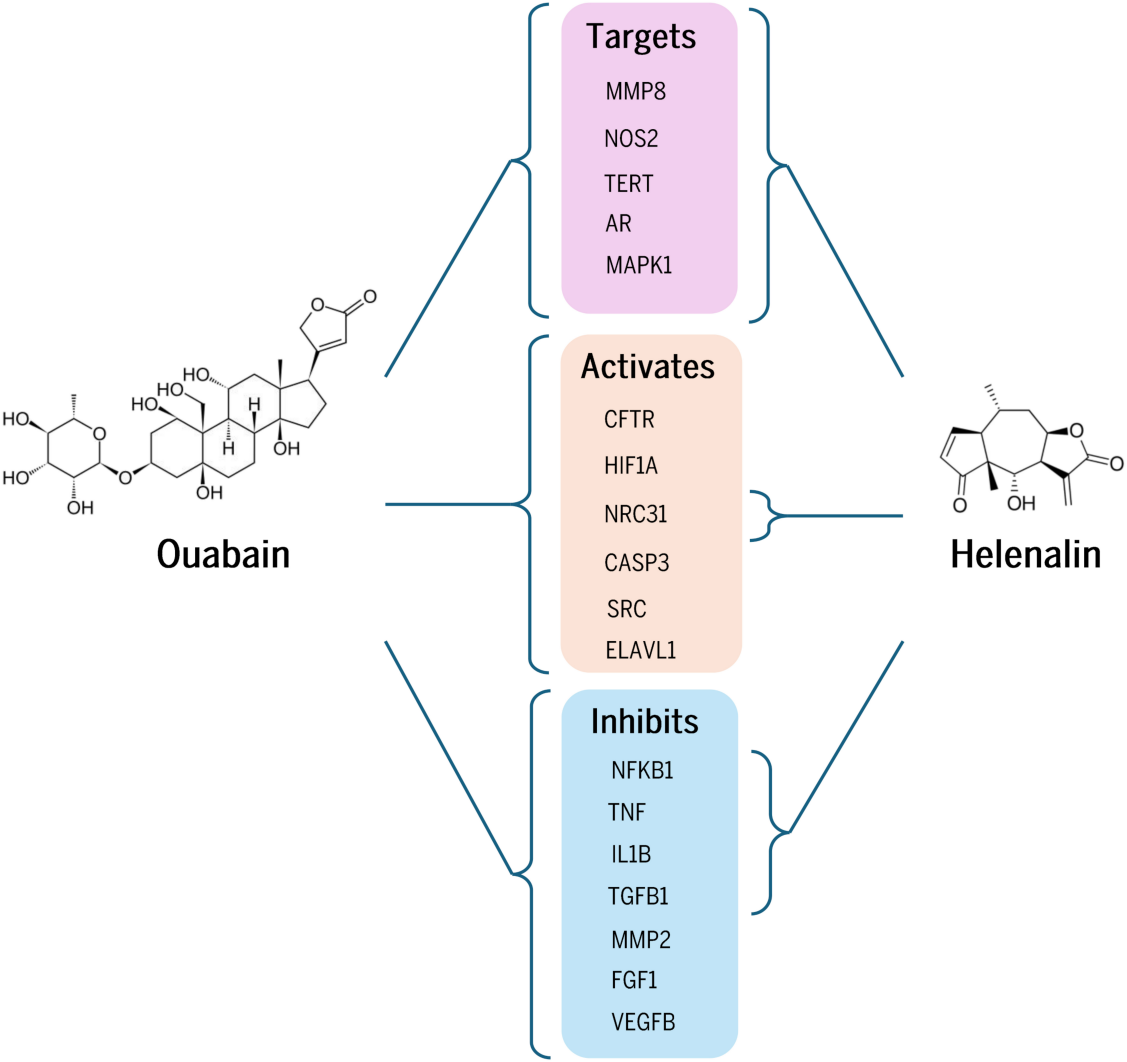
Multi-target pharmacological interactions of ouabain and helenalin. Examples of targeting, inhibiting, and activating interactions between ouabain, helenalin, and genes.

Two of the active compounds from HTS, helenalin and ouabain, showed especially interesting features. The first compound, helenalin, was found to be active in all bioassays. Helenalin is a sesquiterpene lactone, which has been used in traditional medicine as an anti-inflammatory and analgesic agent [31], but it is yet to be used in the clinic. The second compound, ouabain, was found to be active in 4 of 5 bioassays. Ouabain is a cardiac glycoside traditionally utilized for hypotension and arrhythmia [32,33]. Since both compounds are relatively poorly characterized, we utilized LINCS L1000 and SwissTarget to retrieve further information on potential binding targets. Our analysis showed that ouabain and helenalin share several targets, including *NR3C1*, *NOS2*, *TERT*, *MMP2*, and *MMP8.* Additionally, ouabain targets, for example, *TNF*, *FGF*, *VEGF*, *MMP1*, *MMP7*, *MMP9*, *MMP13*, and *MMP14* (Fig. 2 and Table S2)*.* All these genes play pivotal roles in lung fibrosis [2,34,35].

Of the 509,527 unique compounds from bioassays, 2,323 (126 active and 2,206 inactive) (Table S3) were represented in UKS [26–29], which is a powerful modeling tool for data-driven analysis in drug and chemical development providing a structured framework to represent interconnected entities and relationship between chemicals and genes. Our overrepresentation analysis identifying genes within the UKS that were associated more frequently with active compounds than inactive ones found significant “binding” associations with *NR3C1*, *SERPINA6*, *NQO1*, and *NXA1* (Table S4). These genes are more likely bound by the active compounds than the non-active ones. *NR3C1* showed significant binding associations, indicating that NR3C1 is an important target for several compounds active in fibrosis-related pathways. Ouabain was one of the active compounds demonstrating binding to *NR3C1* (Table S2)*.* Helenalin was absent from the UKS, but showed NR3C1 activity in HTS. Together, these observations from HTS, LINCS L1000, SwissTarget, and UKS highlighted especially ouabain and helenalin as promising candidates for further characterization.

### Ouabain, helenalin, and gentamicin C have binding affinities comparable to FDA-approved drug targets

**Fig. 3.**
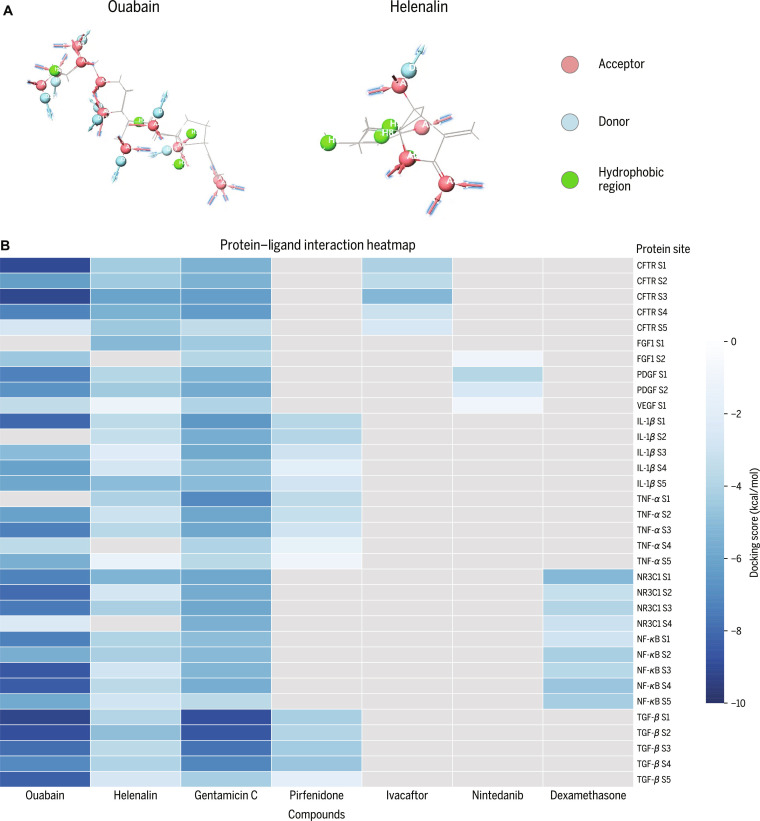
Pharmacophore features and docking scores. (A) Pharmacophore features of ouabain and helenalin. (B) Docking scores of candidates (ouabain, helenalin, and gentamicin C) and FDA-approved drugs (pirfenidone, ivacaftor, nintedanib, and dexamethasone) with key target proteins across potential binding sites (S = site). Gray = not tested.

We next examined the pharmacophoric features of ouabain and helenalin that could underpin their activity (Fig. 3A; full analysis in Fig. S1). These properties were used in virtual screening to identify compounds that resemble helenalin and ouabain’s biological interactions, facilitating discovery of potential analogous treatment candidates and serving as initial filtering. For helenalin, the list of most similar compounds included 2,3- dihydrohelenalin and other helenalin derivatives (Table S5). For ouabain, an array of cardiac glycosides was revealed, including digoxin, acolongifloroside, and desglucocheirotoxin (Table S6). Furthermore, among the highly ranked compounds was gentamicin C, which has attracted considerable interest due to its prospective therapeutic benefits for CF [36]. Therefore, we considered it an addition to the potential candidates in our analysis.

To investigate direct potential binding affinities of ouabain, helenalin, and gentamicin C to target proteins, computational docking was used. First, the optimal binding sites of current FDA-approved therapy targets CFTR, fibroblast growth factor 1 (FGF1), platelet-derived growth factor (PDGF), IL-1β, TNF-α, NR3C1, TGF-β, and NF-κB were identified using binding site detection analysis (Fig. S2A). Next, we quantified ouabain, helenalin, and gentamicin C’s affinities to binding sites of these proteins. Across multiple binding sites, ouabain and gentamicin C showed comparable or greater potential binding affinities to the tested proteins, while helenalin showed moderate docking scores (Fig. 3B). Detailed binding site information is available in Fig. S2B. Neither ouabain nor helenalin triggered pan-assay interference compounds (PAINS) alerts, and both were classified as low risk for colloidal aggregation by Aggregator Advisor. Each carried a single Brenk alert, ouabain’s cardiac glycoside scaffold, and helenalin’s α-methylene-γ-lactone, a Michael acceptor whose electrophilic reactivity is considered when interpreting its bioassay activity. Details of the analysis are presented in Table S7.

### IPF samples differentially express genes targeted by candidate drugs

**Fig. 4.**
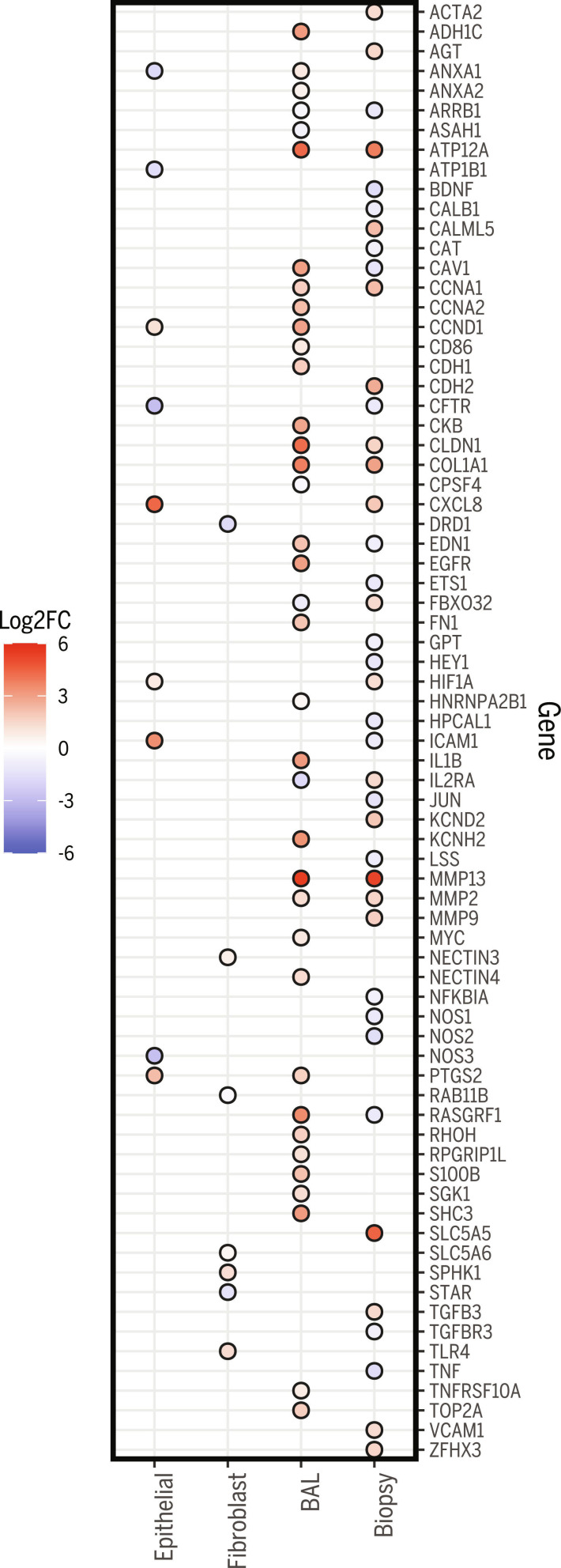
RNA-seq results comparing healthy humans and idiopathic pulmonary fibrosis patients. Data shown for genes targeted by helenalin, ouabain, or gentamicin C. A maximum of 40 differentially expressed genes (*P*_adj_ < 0.05) shown per sample type. BAL, bronchoalveolar lavage. Full data shown in Table S8.

Next, we proceeded to investigate whether the gene targets of ouabain, helenalin, and gentamicin C (from HTS and UKS) were differentially expressed in transcriptomic datasets from human IPF patients to confirm these compound’s therapeutic potential. Genes represented by the HTS bioassays (*TGFBs*, *NFKB*, *CFTR*, *IL1B*, *TNF*), as well as several genes indicative of a progressed state of IPF, such as peptidases (*MMP2*, *MMP9*, *MMP13*), extracellular matrix components (*COL1A1*, *FN1)*, and secreted signaling factors (*CXCL8*, *NOS3*, *JUN*), were indeed dysregulated in IPF patients (Fig. 4, all differentially expressed target genes in Table S8 and original data in [37,38]).

### Helenalin shows an advantageous pharmacokinetic profile

To identify pharmacokinetic properties of our candidate drugs, we performed pharmacokinetic analysis. The absorption, distribution, metabolism, and elimination (ADME) reflect a drug’s pharmacokinetic behavior through different administration routes. Profiling of ouabain, helenalin, gentamicin C, pirfenidone, nintedanib, and ivacaftor revealed marked diversity in their pharmacokinetic behaviors, as highlighted by molecular weight (MW), percent unbound plasma protein, and oral absorption risk (Table 1). In our analyses, gentamicin C and ouabain exhibited high absorption risk scores (5.80 and 5.00, respectively), suggesting potential challenges in oral bioavailability. Conversely, helenalin displayed minimal oral absorption risk score, indicating favorable oral absorption profile. Plasma protein binding was notably high for gentamicin C, but substantially lower for helenalin and ouabain. We also analyzed the compound’s volume of distribution (Vd), referring to a drug’s ability to distribute into tissues. For helenalin, the Vd was 1.432 l/kg and for ouabain 0.799 l/kg, which were lower than, for example, nintedanib’s (4.51 l/kg). For gentamicin C, the value was 0.52 l/kg, indicating low distribution ability (Table 1).

**Table 1.** Description of the pharmacokinetic properties of ouabain, helenalin, gentamicin C, pirfenidone, nintedanib, and ivacaftor

Chemical name	MW (g/mol)	Human unbound plasma protein fraction (%)	Oral absorption risk (score)	Metabolizing CYP enzyme	Volume of distribution (l/kg)	ECCS class	Lipinski’s rule of 5
Ouabain	584.66	59.62	5.00	CYP3A4	0.79	Class 4	Hb, NO, MW
Helenalin	262.30	56.48	0.00	CYP3A4	1.43	Class 2	
Gentamicin C	477.60	95.87	5.80	CYP2B6	0.52	Class 4	Hb, NO
Pirfenidone	185.22	17.14	0.00	CYP2C19	1.26	Class 2	
Nintedanib	539.63	5.92	1.50	CYP2C8	4.51	Class 4	MW
Ivacaftor	392.50	3.95	1.86	CYP2C19	2.64	Class 2	

CYP, Cytochrome P450 enzyme; ECCS, Extended Clearance Classification System; Hb, hydrogen bond donors MW, Molecular weight; NO, number of nitrogen and oxygen atoms

Lipinski’s Rule of Five assesses drug-likeness by evaluating MW, hydrogen bond donors (Hb), and the number of nitrogen and oxygen atoms (NO). As seen in Table 1, helenalin and pirfenidone meet these criteria, indicating favorable absorption and permeability across cell membranes. However, ouabain, gentamicin C, and nintedanib exceed the MW threshold (>500 g/mol) and possess high Hb and NO counts, potentially limiting their oral bioavailability. Pharmacokinetic profiles were also assessed through clearance mechanisms [extended clearance classification system (ECCS)] [39], and metabolism through CYP enzymes. Results of these supporting analyses are presented in Table 1.

Summarizing these findings, ouabain showed comparable ADME profile to ivacaftor and nintedanib; however, the MW, high NO, and oral absorption risk gives ouabain challenging pharmacokinetic properties. Helenalin and pirfenidone had the most suitable ADME profiles, performing well against Lipinski’s rules and demonstrating excellent potential oral bioavailability. As gentamicin C exhibited suboptimal ADME properties (high Hb, NO, and oral absorption risk) and is already in commercial use, we did not further analyze it. While the Lipinski roles provide a useful insight about bioavailability, many approved drugs breach one or more criteria yet remain clinically successful. We therefore interpret these descriptors as flags for further pharmacokinetic assessment rather than as exclusion criteria. The complete ADME analysis is presented in Table S9.

**Fig. 5.**
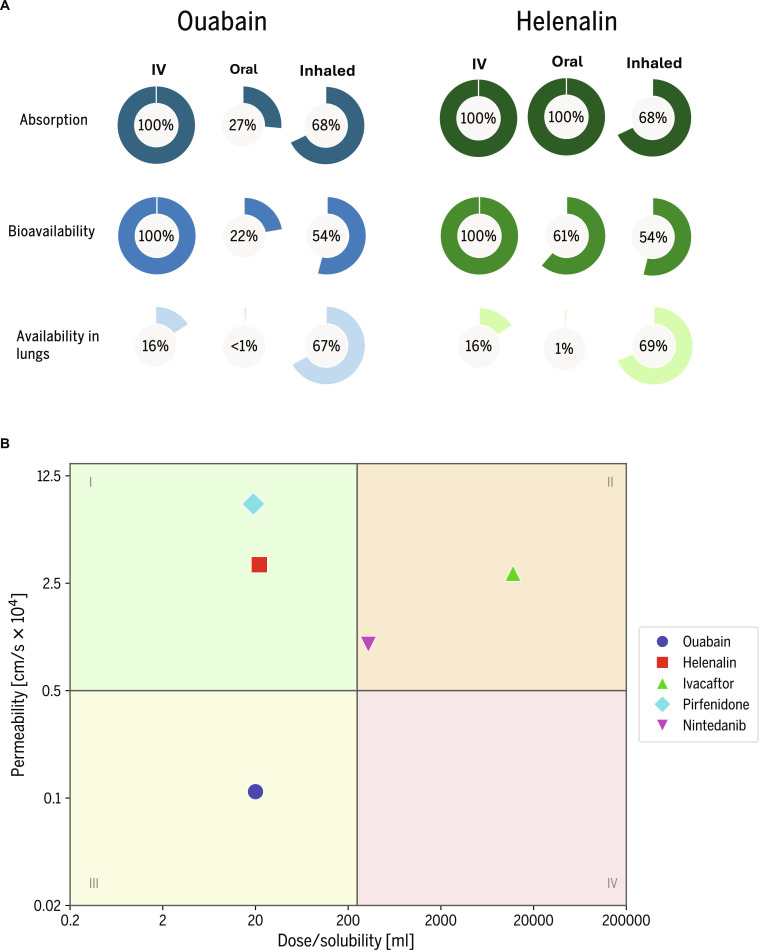
Drug permeability and solubility analysis. (A) Modeled absorption and availability fractions of ouabain and helenalin through different administration routes. (B) Biopharmaceutical classification system analysis for ouabain, helenalin, pirfenidone, nintedanib, and ivacaftor. IV, intravenous

To determine the optimal dosage deliverable to the lungs, we employed exploratory physiologically based pharmacokinetic (PBPK) and biopharmaceutical modeling. We modeled ouabain and helenalin’s absorption and bioavailability fractions via intravenous (IV), oral, and inhaled routes (Fig. 5A). Both compounds showed excellent modeled absorption and bioavailability through IV, whereas corresponding oral values were much lower. Helenalin demonstrated greater potential for oral administration than ouabain. Inhalation absorption and bioavailability values for ouabain and helenalin were very similar (Fig. 5A).

To understand the drug fraction specifically in the lungs, tissue-specific modeling was utilized (Fig. 5A). For ouabain, oral administration resulted in less than 1% lung deposition, IV delivered 16%, and inhalation delivered 67%. For helenalin, oral administration delivered 1%, IV 16%, and inhalation 69% (Fig. 5A). Overall, the pharmacokinetic simulations demonstrated that inhalation clearly enhances drug delivery to the lungs, achieving high local concentration and prolonged half-life of both compounds. Detailed pharmacokinetic modeling supporting these findings is in Fig. S3.

Intestinal epithelial cell membrane permeability and body liquid solubility characteristics of candidates were assessed using the biopharmaceutical classification system (BCS) (Fig. 5B). Our analysis showed that helenalin has better permeability than ivacaftor or nintedanib and a value close to pirfenidone. Between ouabain and helenalin, ouabain has lower permeability but similar solubility values compared to helenalin and pirfenidone. In contrast, ivacaftor and nintedanib had higher solubility than helenalin, ouabain, or pirfenidone.

### In vitro validation of ouabain and helenalin target pathways

To assess cytotoxicity, helenalin, ouabain, and pirfenidone (as positive antifibrotic control) were tested across a range of concentrations, using barrier integrity, cell viability, and RNA yield as indicators. Based on these results (Fig. S4), 3 sublethal concentrations for each compound were selected for subsequent efficacy testing. To evaluate ouabain’s and helenalin’s potential to mitigate the profibrotic effects of TGF-β in human alveolar epithelial cells, we repeatedly exposed the cells to 10 ng/ml TGF-β1, combined with 3 different concentrations of ouabain (5 to 10 nM, T + O_5–10), helenalin (0.5 to 3 μM, T + H_0.5–3), or pirfenidone (0.05 to 0.4 mg/ml, T + P_0.05–0.4) over a period of 5 d.

**Fig. 6.**
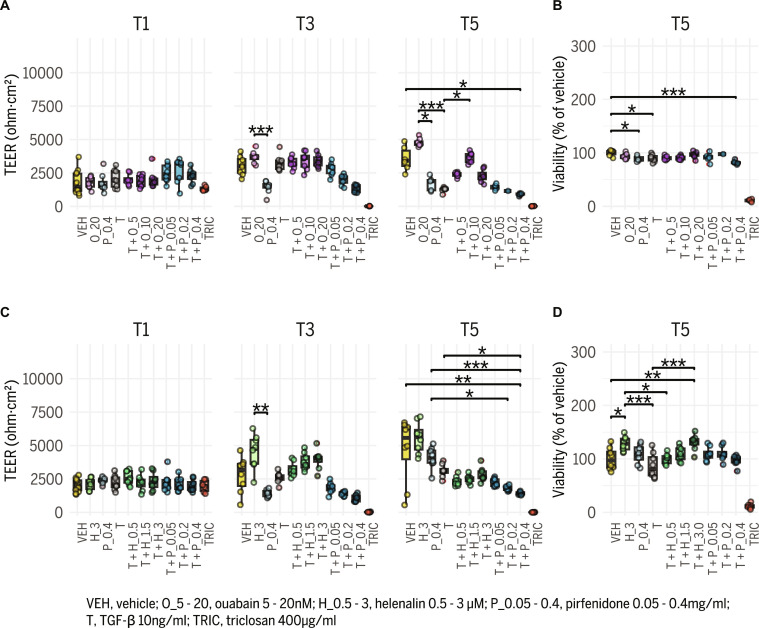
TEER and viability results. Changes in TEER (A and C) and viability (B and D) of huArlo cells after 5 d of TGF-β (T), ouabain (O), helenalin (H), or pirfenidone (P) exposure. *n* = 5 to 8 biological replicates/group except for VEH (T1) *n* = 12 and T + P_0.4 (A, B T5) *n* = 1. Only significant comparisons between vehicle, TGF-β 10 ng/ml, or drug only-groups are shown. *P < 0.05, **P < 0.01, ***P < 0.001.

First, we assessed the impact of these drug–TGF-β combinations on cell barrier function (Fig. 6A and C). The most pronounced effects were observed on days 3 and 5 (full analysis including the effect sizes and confidence intervals in Fig. S5 and Table S10). TGF-β (T) alone had a nonsignificant lowering effect on barrier function. On day 5, the T + O_10 group had a significantly higher transepithelial electrical resistance (TEER) value than the T group (*P* < 0.05). The values of T + O_10 did not differ from the vehicle group (VEH), indicating a protective effect of ouabain on barrier function. In the case of helenalin, none of the groups differed from VEH. In T + P_0.4 groups, TEER value decreased significantly (*P* < 0.05) compared to VEH (Fig. 6A and C). All drug groups except T + P_0.4 on T5 kept their group mean TEER value ≥1,000 Ω*cm^2^, which we considered to indicate a functional barrier.

Repeated TGF-β treatment reduced cell viability by ~10% (Fig. 6B and D). Ouabain alone had no effect compared to VEH, but when combined with TGF-β it reversed the viability drop caused by TGF-β (Fig. 6B). H_3 and T + H_3 showed ~25% higher viability compared to VEH (Fig. 6D). Cell viability in P_0.4 varied slightly between the 2 experimental repeats (Fig. 6B and D). At higher concentrations, pirfenidone caused significant cytotoxicity compared to the other groups, whereas TGF-β alone and all helenalin and ouabain groups had no significant effect on cytotoxicity (Fig. S5 and Table S10). Based on the results from the TEER, viability, and cytotoxicity analyses, we confirmed that we were working at sublethal rather than highly toxic concentrations, representing a more physiologically relevant therapeutic environment.

**Fig. 7.**
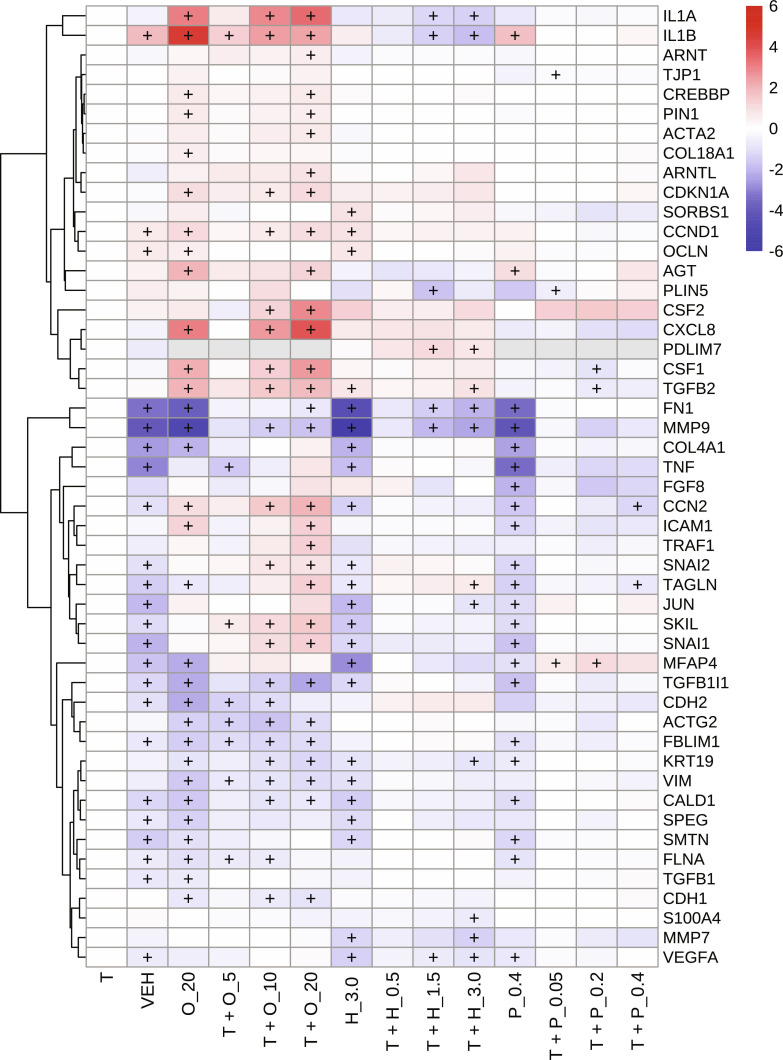
Gene expression after exposures. Log_2_ fold changes (blue-to-red scale) in epithelial cell gene expression after 5 d of TGF-β (T), ouabain (O), helenalin (H), and pirfenidone (P) exposures (*n* = 2 to 4 biological replicates/analyzed group). TGF-β 10 ng/ml (T) was used as the control. Significant (*P*_adj_ < 0.05) changes marked with “+”.

Gene expression after exposures was investigated with a gene panel related to inflammation and epithelial-to-mesenchymal transition (Fig. 7). Compared to VEH, TGF-β treatment (T) induced a proinflammatory response significantly up-regulating *FN1*, *MMP9*, *COL4A1*, and *TNF* while down-regulating *CCND1* and *IL1B*, showing concordance with the fibrotic patient samples (Table S8). When ouabain was administrated with TGF-β (T + O_5 – 20), a down-regulation was observed for *ACTG2*, *FBLIM1*, *VIM*, and *CALD1* and up-regulation for *IL1A*, *IL1B*, and *CXCL*8, resulting in enrichment of apoptotic pathways in a concentration-dependent manner. Increasing helenalin concentrations with TGF-β resulted in down-regulation of *FN1*, *IL1A*, *IL1B*, *MMP7*, *MMP9*, and *VEGFA* compared to T only, suggesting a partial reversal of the effects of TGF-β on these genes (Fig. 7). With pirfenidone, there were fewer significantly altered gene responses compared to ouabain and helenalin. Pirfenidone together with TGF-β caused a concentration-dependent down-regulation of *CCN2 and TAGLN* and up-regulation of *MFAP4*. Full result data are presented in Table S11 and Fig. S7.

## Discussion

In this study, we present a novel data-driven drug discovery workflow that identified promising multi-target therapeutic candidates for lung fibrosis. Our workflow combines high-throughput screening, integrative data modeling, virtual screening, ADME/PBPK prediction, transcriptomics, and in vitro validation to overcome the limitations of single-method drug discovery approaches [40–42]. This approach expands the chemical and biological search space beyond that accessible via conventional HTS alone, enabling identification of novel mechanistic targets in IPF [42]. Moreover, integrating a knowledge graph facilitates the discovery of non-obvious relationships among diverse datasets [43,44]. Related computational efforts have each addressed part of this challenge: Tools such as *drexml* and HerbComb infer mechanistic and synergistic hypotheses from aggregated target and omics data [10,12], network-pharmacology frameworks extend this to multi-scale molecule-to-patient reasoning [11], and fibrosis-specific toxicogenomic and clinical-prediction studies anchor either end of the mechanism-to-outcome spectrum [45]. However, these approaches typically depend on preexisting omics measurements or operate at a single scale, whereas our workflow integrates these dimensions end-to-end within a single pipeline. Our approach offers a transformative paradigm for drug discovery by addressing multiple biological dimensions overlooked by traditional methods [43,44,46,47]. The novelty of this study lies in the integration of multiple complementary layers into a unified prioritization framework.

Besides one study on lung fibrosis [48], limited studies have systematically integrated combinatorial HTS approaches to investigate the mechanistic pathways underlying IPF disease progression. We initiated our workflow (Fig. 1) with an HTS analysis, including relevant lung disease-specific targets and pathways, and identified a pool of potential candidate therapeutics. Identifying compounds active across multiple bioassays is a pivotal advance, as it indicates therapeutic candidates with potential multi-target efficacy in IPF treatment [49]. This is particularly important given the molecular mechanistic complexity of IPF pathology [49,50]. In our investigation, we identified 24 compounds active in fibrosis-associated bioassays. We also identified *NR3C1* as a significant target across bioassays, highlighting its role as a central regulator in pulmonary fibrosis. NR3C1, a widely expressed glucocorticoid receptor, plays a crucial role in modulating myofibroblast activation, extracellular matrix synthesis, and profibrotic signaling pathways [51,52]. This known role of NR3C1 supports both the derivation of active compounds from HTS and the active compound–gene interaction analysis using the UKS.

Combining this with publicly available pharmacological data, ouabain and helenalin emerged as the strongest potential drug candidates. By inhibiting the Na^+^/K^+^-ATPase (adenosine triphosphatase) pump, ouabain perturbs ion homeostasis, triggering downstream signaling cascades involved in fibroblast differentiation and contraction [32,53]. Recent literature suggests that this activity can also influence TGF-β signaling and, by extension, possibly fibrogenesis. While the details in the antifibrotic mechanism remain partially unclear, decreased cell proliferation and increased apoptosis [32], down-regulation of TGF-β receptor 2 and Smad2 phosphorylation [54], and increased prostaglandin expression and protein kinase A activation [53] have been suggested as mechanisms through which ouabain attenuates fibrotic marker expression and collagen deposition in vivo [32]. Moreover, inhaled ouabain has been previously trialed as an asthma treatment, showing effectiveness in mild to moderate asthma [55]. Helenalin, on the other hand, exerts potent anti-inflammatory effects by irreversibly inhibiting NF-κB, thereby suppressing transcription of multiple pro-inflammatory and pro-fibrotic cytokines [31,56–58]. This directly addresses the chronic inflammatory milieu perpetuating fibrosis in IPF, aligning with findings linking NF-κB activity to disease progression [59]. Both ouabain and helenalin were also found to bind *NR3C1*.

Due to their effectiveness in multiple bioassays and predicted binding to *NR3C1*, further pharmacological analysis was continued with ouabain and helenalin, revealing that they exhibited potential binding affinities comparable to the FDA-approved treatments at all investigated binding sites (Fig. 3). Previous drug repurposing approaches have demonstrated the power of docking analysis, for example, by revealing celecoxib and daclatasvir as a potential anti-inflammatory and antifibrotic candidates [60–62]. While important, binding affinity is not always directly associated with drug efficacy toward a specific target [63]. Ouabain’s inhibition of Na^+^/K^+^-ATPase underlies its narrow therapeutic window and known cardiotoxicity, while helenalin’s electrophilic α-methylene-γ-butyrolactone confers reactivity toward off-target nucleophilic residues, raising the risk of nonspecific covalent binding and associated toxicity.

Ouabain’s and helenalin’s potential was explored by investigating lung samples from IPF patients. We confirmed differential expression of several ouabain and helenalin target genes including *CFTR*, *IL1B*, and *TNF* as well as several matrix metalloproteases (MMPs) and extracellular matrix components (Fig. 4). A newly published study integrating single-cell and bulk RNA-sequencing (RNA-seq) data from fibrotic lung tissues applied a conceptually similar approach to ours to retrieve potential drug candidates [64]. However, unlike our workflow, it did not incorporate data-driven target prioritization prior to compound selection nor subsequent in vitro validation.

To better understand the biological properties of ouabain and helenalin, pharmacokinetic analysis was performed. Pharmacokinetics has proven valuable in drug discovery, with recent examples such as the artificial intelligence (AI)-designed kinase inhibitor for IPF, where modeling enabled successful ADME optimization in just 18 months, and subsequent phase I safety validation [65]. In our study, pharmacokinetic data for ouabain and helenalin were absent, so we undertook modeling to simulate their profiles and compare this with FDA-approved treatments. Our analysis is the first to describe the full pharmacokinetic profiles for both compounds and suggested that inhalation is the optimal route (Fig. 5), allowing direct lung absorption while minimizing systemic exposure and associated risks. This aligns with recent research where inhaled formulations of pirfenidone and nintedanib showed promising results in early-phase trials and preclinical models [66–71]. For helenalin, the dosage used here was theoretical (same dosage as ouabain) due to the lack of available pharmacokinetic data, which may limit result interpretation. In addition, although ouabain did not meet optimal ADME criteria, it is noteworthy that several FDA-approved drugs in the comparator set similarly exhibited limitations in solubility and permeability, underscoring that such physicochemical constraints do not necessarily preclude therapeutic utility.

During early fibrosis after injury, several cell types in the lungs start secreting TGF-β, including type II epithelial cells and macrophages [16]. Adequate TGF-β signaling is crucial for wound healing due to its proliferative and angiogenic properties, but excessive TGF-β signaling leads to fibrosis [72]. To assess whether ouabain and helenalin could counteract a prototypical profibrotic response induced by TGF-β, they were administered to TGF-β-treated lung epithelial cells (Fig. 7). Previous in vitro studies show that ouabain down-regulates *CFTR* expression, reduces fibroblast proliferation, and promotes apoptosis [32,33]. In a fibrotic mouse model, ouabain significantly reduced *ACTA2*, *FN1*, and *COL1A1* expression [32]. In contrast, in our model, ouabain decreased *FN1* and *VIM* expression, but did not affect *CFTR* or *COL1A1* expression, and in the highest tested concentration, *ACTA2* expression increased. In addition to the partial absence of metabolic enzymes in vitro, ouabain has been shown to exert either pro- or anti-inflammatory effects depending on the administration route, concentration, and involvement and activation status of the immune system [73], which may explain these discrepancies between our findings and previous in vivo studies. In vivo, helenalin treatment down-regulates fibrotic markers in the liver, including *COL4A1*, *ACTA2*, and *MMP9* expression [58]. Consistent with these previous findings, our study also demonstrated that helenalin significantly reduced the expression levels of *MMP9* and *FN1* in addition to *IL1A* and *IL1B* compared to cells treated with TGF-β alone.

Overall, the gene expression patterns retrieved were consistent across concentrations. Helenalin treatment alone showed a profile similar to vehicle, and when combined with TGF-β, helenalin was able to partially revert the expression changes caused by TGF-β back toward a more vehicle-like profile. Helenalin’s profile predominantly exhibited an anti-inflammatory response, whereas ouabain exposure was characterized with both anti- and pro-inflammatory responses. This pro-inflammatory response was further amplified when increasing ouabain concentrations were administered with TGF-β. The expression pattern related to ouabain possibly suggested an activation of NF-κB and apoptotic pathway in a concentration-dependent manner, due to up-regulation of cytokine transcripts such as *IL1A*, *IL1B*, and *CXCL8*, and a partial down-regulation of epithelial–mesenchymal transition (EMT)-related processes, whereas for helenalin the gene expression changes pointed toward EMT suppression. Pirfenidone, on the other hand, showed both pro-inflammatory and anti-inflammatory expression changes with a slightly different pattern compared to ouabain and helenalin. This further suggests that ouabain and helenalin might have beneficial properties compared to current therapies such as pirfenidone. While the results concerning ouabain seem promising, ouabain has previously been associated with cardiotoxic and inflammatory effects, and therefore, therapeutic window might be very narrow [74,75]. The cardiotoxic and cardiotonic effects are caused by the inhibition of Na^+^ release, calcium accumulation, and increased heart muscle contraction. When ouabain is administered via inhalation, the primary exposed cell types are epithelial cells, where Na/K-ATPase function is known to control lung fluid clearance [76]. Although potential side effects must be considered carefully, there is evidence that concentration plays a critical role in determining the biological response. At low concentrations, ouabain can induce positive effects, such as epithelial barrier strengthening [77] and decreased airway inflammation [33]. Thus, systemic side effects may be more controllable when ouabain is administered via inhalation because lower concentrations can be used. Due to the conflicting data regarding ouabain safety, further fibrosis-specific research is warranted before concluding its potential as a new therapeutic agent.

While alveolar epithelial cells are widely considered as the initiators of events leading to pulmonary fibrosis, using a single cell line model to investigate the effects limits the generalizability of the in vitro results. Thus, further assessment of the results is needed with a model consisting of other fibrosis-related cell types too. In addition, protein level investigation of the functional endpoints (e.g., collagen deposition and extracellular matrix production) supporting transcriptional findings would strengthen the analysis further.

## Conclusions

This study demonstrated the power of integrating bioassays, public databases, docking analyses, and pharmacokinetics to accelerate the identification of new therapeutic candidates for pulmonary fibrosis. Ouabain and helenalin emerged as promising drugs with significant activity across fibrosis-associated pathways, comparable to FDA-approved treatments. Their ability to target multiple fibrosis-associated targets simultaneously may be more effective in addressing the multifaceted nature of fibrotic pathologies compared to current treatments. Moreover, both compounds showed good potential bioavailability, and in alveolar epithelial cells, especially helenalin showed a strong ability to regulate profibrotic gene expression. Helenalin was also safer in ADME analysis, suggesting that it is the most favorable candidate for follow-up assessments. The approach presented here is applicable beyond pulmonary fibrosis and can extend to other complex pathologies, paving the way for improved patient outcomes across diseases.

While these computational approaches provide valuable mechanistic insights, they are inherently limited and cannot fully capture the complexity of IPF pathobiology. The limited availability of experimental pharmacokinetic and pharmacodynamic data further constrained mechanistic interpretation and precluded a comprehensive characterization of the compounds’ pharmacological behavior. Additionally, compounds exhibiting strong binding affinity across a broad range of molecular targets may raise concerns regarding selectivity, as promiscuous binding profiles are often associated with an increased risk of off-target toxicity. Furthermore, more advanced structural analyses including molecular dynamics simulations, MM-GBSA (molecular mechanics–generalized Born surface area) rescoring, and induced-fit docking were not performed in this study and would, in future work, help refine the predicted binding poses and further validate the docking-derived results. These limitations highlight the need for future experimental validation.

## Methods

### Data collection for potential candidate molecules

Five lung fibrosis relevant bioassays were selected for analysis: CFTR correctors (PubChem AID 720511), NF-κB inhibitors (PubChem AID 588850), IL1B inhibitors (PubChem AID 743279), TGF-β inhibitors (PubChem AID 588855), and TNF-α inhibitors (PubChem AID 463075). The detailed information related to assays is provided in Table S12. The selection of the bioassays was based on the signaling molecule’s contribution to lung fibrosis and their role as targets of FDA-approved treatments nintedanib, pirfenidone, ivacaftor, and dupilumab [2–4]. The activity classification for each compound was based on the active/inactive labels assigned within each bioassay as reported in PubChem, which reflect each assay’s own criteria for inhibition or activation. Transcriptional profiles induced by ouabain treatment were analyzed using the LINCS L1000 platform (Touchstone v1.1) [78,79]. We utilized ouabain-specific signatures from cell lines A375, A549, MCF7, and PC3 [80]. Target predictions from SwissTargetPrediction were also incorporated into analysis, although this source carries inherently lower confidence relative to experimentally curated databases given its probabilistic nature.

In-house knowledge graph UKS [26–29] was used to extract compound–gene interactions. Specifically, we compiled data for connections where a drug (a) activates (TransmiR and STITCH), (b) inhibits (STITCH and DrugBank), (c) binds (STITCH and DrugBank), (d) targets (DrugBank, Open Targets, and Pharos), or (e) affects a gene (DrugBank, Pharos, and The Comparative Toxicogenomics Database). To assess the statistical significance of UKS-derived compound–gene interactions, Fisher’s exact tests were performed comparing each gene’s association frequency with active versus inactive compounds. Genes with a false discovery rate (FDR)-corrected *P* value below 0.05 were considered significantly enriched in active compounds. We used the data layer of UKS in the analysis.

The candidate molecule selection was based on the following criteria: HTS activity across ≥4/5 assays as primary filter; UKS as primary target retrieval source; LINCS L1000 and SwissTarget as independent orthogonal validators.

### Molecular structures and ligand preparation

SMILES representations were converted into 2-dimensional (2D) molecular structures using the 2D Sketcher in Maestro molecular modeling package. For the ligand preparation in 3D, LigPrep in Maestro suite was executed, neutral pH was used in the OPLS4 force field, and the maximum ligand size was 500 atoms. Chirality was determined based on 3D configurations while keeping other parameters at their default values [81,82]. The Compare Ligand Files tool (Maestro) was used to compare the ligands between bioassays.

### Pharmacophore models and virtual screening

Pharmacophore models define the key 3D arrangement of chemical features required for target binding. Virtual screening uses these models to rapidly identify likely active candidates from large compound libraries in silico. Maestro’s Phase tool was employed to identify mutual features and substructures among active ligands, assisting in constructing a unified pharmacophore model. This model was then used for virtual screening against a compound library from the UKS, prepared and converted to 3D structures. Phase’s Ligand and Database Screening tool was employed to perform the virtual screening for ouabain and helenalin based on their matching to pharmacophore model for each compound. The scoring phase score was used to assess how compounds from virtual screening matched the pharmacophore model and their fitting to the 3D models. Additional conformers were not assigned while keeping other parameters at their default values [83].

### Binding site detection and molecular docking simulations

Binding site detection and molecular docking simulations identify likely ligand-binding pockets on a protein and predict the preferred orientation and affinity with which a small molecule fits into them, providing structural hypotheses for compound–target interaction. Targets for docking (CFTR, FGF1, FGF2, IL-1β, NR3C1, VEGFA, PDGF, TNF-α, TGF-βRI) were retrieved from Protein Data Bank and UniProt, and their corresponding protein structures (5UAK, Q6I6M7, P09038, 1ITB, 7KW7, AF-P15692-F1, AF-P04085-F1, 2AZ5, and 1B6C) were subsequently employed. For NF-κB, Matthias Stein’s model was used due to the lack of the full protein structure in PDB [84]. While AlphaFold-predicted models were employed to ensure full protein structure coverage, this approach is inherently limited by the absence of experimentally resolved structures for several targets. Any absent side chains, backbone atoms, and loop structures were rectified using Prime (Maestro*)*. To ascertain protonation states, structural refinement, and energy minimization, the converge heavy atoms to root mean square deviation 0.3 Å, we enlisted the PROPKA tool and the OPLS-2005 force field, respectively, while keeping other parameters at their default values [85,86]. SiteMap (Maestro) was used to find the best binding sites for each protein, with a setting of 15 site points per reported site, and crop site maps at 4 Å from the nearest site point parameters [85,86]. Maestro’s Glide [extra precision (XP)] was employed to execute the molecular docking scores and binding postures between ligands and target proteins. The configurations obtained during the docking were categorized according to the Glide score function [85,86].

### Structural alert estimation

Candidate compounds were screened for PAINS to flag substructures associated with frequent assay interference and nonspecific bioactivity [87,88]. PAINS filtering was performed using the Baell and Holloway structural alert set (as implemented in RDKit/SwissADME) [87,88], and any matching substructural alerts were recorded for each compound. Colloidal aggregation risk was assessed for candidates using Aggregator Advisor (Shoichet laboratory), with structural similarity to known aggregators used to assign a risk category [89].

### Predicting ADME characteristics

The ligand’s ADME traits were ascertained using the ADMET Predictor v11 (AP11, Simulations Plus Inc., Lancaster, CA, USA) [64]. The simulation parameters were obtained from literature and simulated data, as detailed in Fig. S3.

### In silico PBPK modeling

In silico PBPK modeling uses mathematical representations of the body’s tissues, blood flows, and physiological parameters to mechanistically predict how a drug is absorbed, distributed, metabolized, and excreted over time. GastroPlus (version 9.8.3 and 10.1, Simulation Plus, Lancaster, CA, USA) was used to develop and verify the PBPK models. For the model representing a healthy 30-year-old American male, simulation parameters were derived from literature and simulated data, as detailed in Fig. S3. The ADMET Predictor module was used to obtain in silico estimates of key physicochemical parameters from structures when experimental or clinical values were not available or to provide an objective alternative to experimental data. The metabolism module was used to account for the saturable metabolism of ouabain and helenalin. The advanced compartmental absorption and transit (ACAT) model was used to simulate dissolution and absorption data (oral, IV, inhaled) [90]. The doses were selected based on previously reported human clinical exposure data for ouabain [55,91]. For IV models, the dose was 0.5 mg/ml, for oral 8 mg, and for inhaled 10 mg/ml. For helenalin, we applied the same doses for modeling as for ouabain due to the lack of specific pharmacokinetic data. The simulation parameters were obtained from literature and simulated data and are detailed in Fig. S3.

### IPF patient sequencing data

RNA-seq data to validate the presence of helenalin and ouabain target genes was analyzed from human lung epithelial cells and fibroblasts, bronchoalveolar lavage (BAL), and whole biopsies of IPF patients [37,92]. Transcriptomics data were collected from the Gene Expression Omnibus database. The quality control of sequencing data was performed with FastQC. Trimming of the reads was carried out with Cut adapt [93], and alignment was performed with Hisat2 with GRCh38/hg38 as a reference genome [94]. Counting the reads into genomic features was carried out with feature Counts [95]. For RNA-seq, differential gene expression analyses and normalization of expression matrices were performed with DeSEQ2 [96] and meta-analysis using the MUUMI framework [97–99]. A summary of the datasets is presented in Table S13.

### Cell culture

Human alveolar epithelial cells CI-huArlos (sex = male, p8, #INS-CI-1031, Inscreenex, Braunschweig, Germany) were used to study the effects of helenalin, ouabain, and pirfenidone. Cells were seeded to the apical side of 0.4-μm inserts (#PTHT12H48, Millicell) at 112,000 cells/insert and cultured in liquid-covered conditions (#INS-ME-1013-500, Inscreenex). TEER was measured daily starting day 6 post-seeding (EVOM Manual and STFX4 electrode, WPI, FL, USA) and before the media changes. TEER values are reported as area-normalized.

On day 9 or 11 post-seeding, once TEER had stabilized to ≥1,000 Ω, the exposures were started. Helenalin (#374000-500UG, EMD Millipore, Darmstadt, Germany), ouabain (#O3125-1G, Sigma-Aldrich, Darmstadt, Germany), and pirfenidone (#P2116-50MG, Sigma-Aldrich) were applied alone or with 10 ng/ml TGF-β1 (#T7039-50UG, Sigma-Aldrich). Exposures were given daily to basolateral medium in 40-μl volume. Fresh medium was changed every 2 d before exposures. Vehicle treatment consisted of regular growth medium and solvents dimethyl sulfoxide (DMSO) and phosphate-buffered saline (PBS). Triclosan (Irgasan) (400 μg/ml, #72779, Sigma-Aldrich) served as the positive cytotoxicity control. Sample localization in plates was randomized.

For initial cytotoxicity tests, sublethal concentrations of helenalin, ouabain, and pirfenidone were investigated. Helenalin concentrations up to 20 μM, ouabain 10,000 nM, and pirfenidone 0.6 mg/ml (3.25 mM) were tested with a repeated exposure protocol for 5 d. Based on barrier integrity, RNA yield, and pro-inflammatory gene expression data (Fig. S4), final concentrations of 5, 10, and 20 nM for ouabain; 0.5, 1.5, and 3 μM for helenalin; and 0.05 mg/ml (0.27 mM), 0.2 mg/ml (1.1 mM), and 0.4 mg/ml (2.2 mM) for pirfenidone were used for validation. For TGF-β, concentrations of 5 and 10 ng/ml were tested in the preliminary tests. Five days of daily 5 or 10 ng/ml induced sufficient sublethal profibrotic gene expression response (Fig. S6). We selected the higher concentration for validation.

Exposure effects on cell viability (1:1,000, RealTime-Glo MT Cell Viability Assay, Promega, Madison, WI, USA) and cytotoxicity (1:1,000, CellTox Green Cytotoxicity Assay, Promega) were measured on exposure day 5 according to the manufacturer’s instructions. Reagents were used in both apical and basolateral medium compartments.

Helenalin and ouabain were analyzed in separate experiments, each including identical vehicle, TGF-β (10 ng/ml), pirfenidone, and triclosan control groups. All groups included 5 to 8 biological replicates, except VEH, where *n* = 12 at T1, and T + P_0.2 at T5 in the ouabain experiment, where only one sample remained after exclusion of samples that did not meet quality criteria.

To compare group-wise differences in TEER, viability and cytotoxicity measurement normality was assessed based on the results: Either Kruskall–Wallis + Dunn or analysis of variance (ANOVA) + Tukey was used in R with relevant packages. *P* < 0.05 was regarded as statistically significant. Effect sizes and 95% confidence intervals were analyzed with the R package Effectsize.

### RNA extraction and high-throughput polymerase chain reaction

Cell lysate RNA was extracted from the same experiments in which TEER, viability, and cytotoxicity were assessed, using the RNeasy Mini Kit (Qiagen) with deoxyribonuclease I treatment. Each group included 2 to 4 biological replicates, except in the ouabain experiment, where the final sample size after exclusion of low-quality samples was *n* = 0 for T + P_0.2 and *n* = 1 for P_0.4 and T + P_0.4. cDNA was synthesized (High-Capacity cDNA Reverse Transcription Kit, Applied Biosystems, Waltham, MA, USA) and used as a template for BioMark HD (Fluidigm, South San Francisco, CA, USA). Samples were analyzed as singles. Altogether, 88 primer pairs were used as targets. The gene panel was aimed to specifically target several inflammation and epithelial–mesenchymal transition-related genes, selected based on our previous work [100] and additional manual curation. From the retrieved expression data (Fluidigm Real-Time PCR Analysis v. 4.3.1 with automated Ct thresholding and manual checking of nontemplate controls), genes/groups with only 1 Cq value and outliers were removed [outlier defined as any Cq value either below Q1 − 1.5 * IQR (interquartile range) or above Q3 + 1.5 * IQR]. From the remaining samples (*n* = 2 to 4 biological replicates per analyzed group), log_2_ fold change values were calculated using a custom R script including GeNorm selection for reference genes and compared with the reference group (TGF-β 10 ng/ml). For the polymerase chain reaction (PCR) data, initial statistical significance was assessed with ANOVA and Tukey. Results were corrected with Benjamini–Hochberg correction, and FDR-corrected *P* value <0.05 was regarded as statistically significant. Pathway enrichment analysis was performed using the R package ClusterProfiler on differentially expressed genes from all drug concentration groups, with the PCR panel genes used as the background gene set. Details of the targets and detailed PCR run data can be found from Table S11.

## Data Availability

The datasets supporting the conclusions of this article are provided within the main article and supplementary files. The complete code and data are also available in a Zenodo repository at https://doi.org/10.5281/zenodo.17116952.
