# Supplementary material for: Integrated Approaches in Drug Repositioning Highlight Ouabain and Helenalin as Potential Drug Candidates for Pulmonary Fibrosis
Source: Comput Struct Biotechnol J. 2026 Aug 10;35(1):0193. doi: 10.34133/csbj.0193 (PMC13454465; doi:10.34133/csbj.0193)
Supplement: Supplementary file 1 [file csbj.0193.f1.zip › Al_Abdulraheem et al. Supplementary_files_Figures_reviewed.docx]

Supplementary Figures for Integrated Approaches in Drug Repositioning Highlight Ouabain and Helenalin as Potential Drug Candidates for Pulmonary Fibrosis

*Zeyad Al-Abdulraheem†, Hanna-Kaarina Juppi †, Jack Morikka, Simo Inkala, Noora Perho,* *Saara Sani, Angela Serra, Antonio Federico, Michele Fratello, Dario Greco**

† These authors contributed equally.

* Author to whom correspondence should be addressed ([dario.greco@tuni.fi](mailto:dario.greco@tuni.fi)).

**Supplementary Figure 1.**

Ouabain possesses a lactone ring and hydroxyl groups, with hydrogen donors (in red, Figure S1), such as -OH groups, contributing to hydrogen bonding. Ouabain’s hydrogen acceptors (in blue, Figure S1) include the oxygen atoms of the lactone ring, and it has a hydrophobic region (in green, Figure S1) around its steroidal backbone. Helenalin features an electrophilic α-methylene-γ-lactone moiety that acts as a hydrogen acceptor (in blue, Figure S1), along with hydroxyl groups as hydrogen donors (in red), and hydrophobic regions in its terpenoid framework (in green, Figure S1).

.

A

= Binding site

| **Protein + binding site** | **Pirfenidone**  **(**kcal/mol) | **Ivacaftor**  **(**kcal/mol | **Nintedanib**  **(**kcal/mol | **Dexa-methasone**  **(**kcal/mol | **Ouabain**  **(**kcal/mol | **Helenalin**  **(**kcal/mol | **Gentamicin C**  **(**kcal/mol) |
| --- | --- | --- | --- | --- | --- | --- | --- |
| **CFTR S1** |  | -4.088 |  |  | -8.990 | -4.319 | -5.376 |
| **CFTR S2** |  | -3.628 |  |  | -6.236 | -4.446 | -5.374 |
| **CFTR S3** |  | -5.183 |  |  | -9.140 | -6.027 | -6.265 |
| **CFTR S4** |  | -3.107 |  |  | -7.242 | -5.448 | -6.351 |
| **CFTR S5** |  | -2.628 |  |  | -2.692 | -4.530 | -3.450 |
| **FGF1 S1** |  |  |  |  |  | -5.111 | -4.420 |
| **FGF1 S2** |  |  | -1.013 |  | -4.554 |  | -3.845 |
| **PDGF S1** |  |  | -3.848 |  | -7.276 | -3.839 | -5.281 |
| **PDGF S2** |  |  | -2.581 |  | -6.747 | -4.409 | -5.739 |
| **VEGF S1** |  |  | -0.917 |  | -3.446 | -1.194 | -4.019 |
| **IL1B S1** | -3.818 |  |  |  | -8.078 | -3.573 | -6.489 |
| **IL1B S2** | -3.888 |  |  |  |  | -3.284 | -5.591 |
| **IL1B S3** | -2.993 |  |  |  | -4.974 | -2.180 | -5.759 |
| **IL1B S4** | -1.878 |  |  |  | -6.139 | -2.709 | -4.787 |
| **IL1B S5** | -2.833 |  |  |  | -5.911 | -4.934 | -4.966 |
| **TNF S1** | -3.526 |  |  |  |  | -4.043 | -7.066 |
| **TNF S2** | -3.268 |  |  |  | -6.169 | -3.013 | -5.933 |
| **TNF S3** | -2.947 |  |  |  | -7.351 | -3.746 | -5.885 |
| **TNF S4** | -1.497 |  |  |  | -3.606 |  | -4.020 |
| **TNF S5** | -0.978 |  |  |  | -5.545 | -1.307 | -3.693 |
| **NR3C1 S1** |  |  |  | -5.145 | -7.237 | -5.326 | -5.892 |
| **NR3C1 S2** |  |  |  | -3.270 | -7.943 | -2.704 | -5.718 |
| **NR3C1 S3** |  |  |  | -3.931 | -7.531 | -4.149 | -5.883 |
| **NR3C1 S4** |  |  |  | -3.078 |  | -2.443 | -5.490 |
| **NFKB S1** |  |  |  | -2.942 | -7.308 | -3.955 | -4.915 |
| **NFKB S2** |  |  |  | -4.192 | -5.559 | -4.143 | -5.044 |
| **NFKB S3** |  |  |  | -3.765 | -8.545 | -2.855 | -5.223 |
| **NFKB S4** |  |  |  | -4.598 | -8.469 | -3.663 | -5.622 |
| **NFKB S5** |  |  |  | -4.267 | -5.808 | -2.858 | -3.639 |
| **TGFB S1** | -4.193 |  |  |  | -9.143 | -3.903 | -8.801 |
| **TGFB S2** | -3.930 |  |  |  | -8.800 | -4.871 | -8.566 |
| **TGFB S3** | -4.357 |  |  |  | -7.881 | -3.646 | -7.764 |
| **TGFB S4** | -4.597 |  |  |  | -7.007 | -3.986 | -7.117 |
| **TGFB S5** | -1.872 |  |  |  | -8.246 | -2.673 | -4.223 |
| **FGFR1** |  |  | -5.323 |  | -5.280 | -5.869 |  |
| **FGFR2** |  |  | -4.209 |  | - 3.987 | -4.329 |  |
| **FGFR3** |  |  | - 3.639 |  | -1.880 | - 3.923 |  |
| **Na/K-ATPase** |  |  |  |  | -2.283 |  |  |
| **NFKBp65** |  |  |  |  |  | -4.143 |  |
| **PDGFR A** |  |  | -5.259 |  | -3.250 | - 3.959 |  |
| **PDGFR b** |  |  | - 4.108 |  | -1.940 | - 4.546 |  |
| **VGFR1** |  |  | - 5.229 |  | -6.123 | - 3.715 |  |
| **VGFR2** |  |  | 0.054 |  | - 3.849 | - 4.505 |  |
| **VGFR3** |  |  | -5.292 |  | -4.414 | -5.409 |  |

B

**Supplementary Figure 2.** Optimal binding sites of key fibrotic proteins and docking analysis for selected drugs. A) Optimal binding sites for CFTR, TGF-βR1, NR3C1, TNF-α, IL1β, PDGF, VEGF, and NFKB. B) Full results of the binding analysis for pirfenidone, ivacaftor, nintedanib, dexamethasone, ouabain, helenalin and gentamicin C

**Elimination – Ouabain IV**

**Absorption & Dissolution – Ouabain IV**

**Systemic Distribution – Ouabain IV**

**Plasma Concentration – Ouabain IV**

**A**

**Supplementary Figure 3.** The full results of PBPK models and the used parameters. A) Ouabain, IV B) Helenalin, IV C) Ouabain, oral D) Helenalin, oral E) Ouabain, inhalation F) Helenalin, inhalation

**Supplementary Figure 3.** The full results of PBPK models and the used parameters. A) Ouabain, IV B) Helenalin, IV C) Ouabain, oral D) Helenalin, oral E) Ouabain, inhalation F) Helenalin, inhalation

**Plasma Concentration – Helenalin IV**

**Absorption and Dissolution – Helenalin IV**

**B**

**Systemic Distribution – Helenalin IV**

**Elimination – Helenalin IV**

**Supplementary Figure 3.** The full results of PBPK models and the used parameters. A) Ouabain, IV B) Helenalin, IV C) Ouabain, oral D) Helenalin, oral E) Ouabain, inhalation F) Helenalin, inhalation

**C**

**Plasma Concentration – Ouabain oral**

**Regional Absorption – Ouabain oral**

**Systemic Distribution – Ouabain oral**

**Elimination – Ouabain oral**

**Supplementary Figure 3.** The full results of PBPK models and the used parameters. A) Ouabain, IV B) Helenalin, IV C) Ouabain, oral D) Helenalin, oral E) Ouabain, inhalation F) Helenalin, inhalation

**D**

**Plasma Concentration – Helenalin oral**

**Systemic Distribution – Helenalin oral**

**CpTime – Helenalin oral**

**Regional Absoption– Helenalin oral**

**Elimination – Helenalin oral**

| **Category** | **Variable** | **Value** |
| --- | --- | --- |
| PK Output | Fa(%) | 54.304 |
| PK Output | FDp(%) | 0.246 |
| PK Output | F(%) | 54.244 |
| PK Output | Cmax (µg/mL) | 0.277 |
| PK Output | AUC₀–t (µg·h/mL) | 3.801 |
| PK Output | AUC₀–t (µg·h/mL) | 3.801 |
| PK Output | Cmax Liver (µg/mL) | 1.711 |
| Compartmental Absorption | Pulmonary (%) | 47.6 |
| Lung PBPK Compartments | Extra-thoracic (%) | 18.31 |
| Lung PBPK Compartments | Thoracic (%) | 8.44 |
| Lung PBPK Compartments | Bronchiolar (%) | 13.59 |
| Lung PBPK Compartments | Alveolar-Interstitial (%) | 34.93 |

**Simulation Output – Ouabain inhalation**

**Plasma Concentration– Ouabain inhalation**

**Compartmental Absorption – Ouabain inhalation**

**E**

**Supplementary Figure 3.** The full results of PBPK models and the used parameters. A) Ouabain, IV B) Helenalin, IV C) Ouabain, oral D) Helenalin, oral E) Ouabain, inhalation F) Helenalin, inhalation

| **Category** | **Variable** | **Value** |
| --- | --- | --- |
| PK Output | Fa(%) | 58.23 |
| PK Output | FDp(%) | 1.37 |
| PK Output | F(%) | 57.93 |
| PK Output | Cmax (µg/mL) | 2.054 |
| PK Output | AUC₀–t (µg·h/mL) | 2.2218 |
| PK Output | AUC₀–t (µg·h/mL) | 2.2218 |
| PK Output | Cmax Liver (µg/mL) | 1.220 |
| Compartmental Absorption | Pulmonary (%) | 68.5 |
| Lung PBPK Compartments | Extra-thoracic (%) | 17.68 |
| Lung PBPK Compartments | Thoracic (%) | 7.02 |
| Lung PBPK Compartments | Bronchiolar (%) | 15.06 |
| Lung PBPK Compartments | Alveolar-Interstitial (%) | 32.49 |

**Simulation Output – Helenalin inhalation**

**Plasma Concentration– Helenalin inhalation**

**Compartmental Absorption – Helenalin inhalation**

**F**

**Supplementary Figure 3.** The full results of PBPK models and the used parameters. A) Ouabain, IV B) Helenalin, IV C) Ouabain, oral D) Helenalin, oral E) Ouabain, inhalation F) Helenalin, inhalation

**Supplementary Figure 4.** Results of the TEER, RNA yield and RNA expression analysis of helenalin, ouabain and pirfenidone dose-finding studies. **A- B)** TEER and RNA yield. Exposures were given after TEER measurements on days 1 and 3 (T1 and T3). RNA was collected at day 5 after starting exposures (T5). **C-D)** TEER and RNA yield in the second test where helenalin, ouabain and pirfenidone were administered daily **E)** PCR results from the second test (only significant results are shown). For A and C the unadjusted p-values are presented for relevant comparisons. *p<0.05, **p<0.01, ***p<0.001

**Supplementary Figure 5.** Full results of the TEER, viability and cytotoxicity measurements and statistics related to Figure 6. **A, C)** TEER, and **B,D**) cytotoxicity measurements

**Supplementary Figure 6.** Results of the dose-range-optimization study of TGF-B exposure. A) The effect of daily TGF-B exposures on huArlo barrier. Unadjusted statistics are shown between all other groups except triclosan. B) The effect of repeated TGF-B exposure on huArlo cell viability C-D) The effectof daily TGF-B exposures on huArlo RNA expression after four days (T5) and six days (T7). *p<0.05, **p<0.01, ***p<0.001

**Supplementary Figure 7. A)** Full analysis results related to Figure 7. Heatmap presents all analysed log2Fold values from the data. Blue color indicates downregulation and red upregulation of a gene. Differentially expressed genes (p-adj <0.05 (FDR)) marked with “+”. Grey = no data. **B)** Gene ontology analysis of differentially expressed genes (p-adj <0.05 (FDR) from the TGF-β 10ng/ml and ouabain groups. Other groups did not result in enriched pathways.
