# Supplementary material for: Integrated Approaches in Drug Repositioning Highlight Ouabain and Helenalin as Potential Drug Candidates for Pulmonary Fibrosis
Source: Comput Struct Biotechnol J. 2026 Aug 10;35(1):0193. doi: 10.34133/csbj.0193 (PMC13454465; doi:10.34133/csbj.0193)
Supplement: Supplementary file 1 [file csbj.0193.f1.zip › Al_Abdulraheem et al. Supplementary_files_Figures_reviewed.pdf]

Supplementary Figures for Integrated Approaches in Drug Repositioning Highlight Ouabain and Helenalin as Potential Drug Candidates for Pulmonary Fibrosis

*Zeyad Al-Abdulraheem†, Hanna-Kaarina Juppi †, Jack Morikka, Simo Inkala, Noora Perho, Saara Sani, Angela Serra, Antonio Federico, Michele Fratello, Dario Greco**

† These authors contributed equally.

* Author to whom correspondence should be addressed (dario.greco@tuni.fi).

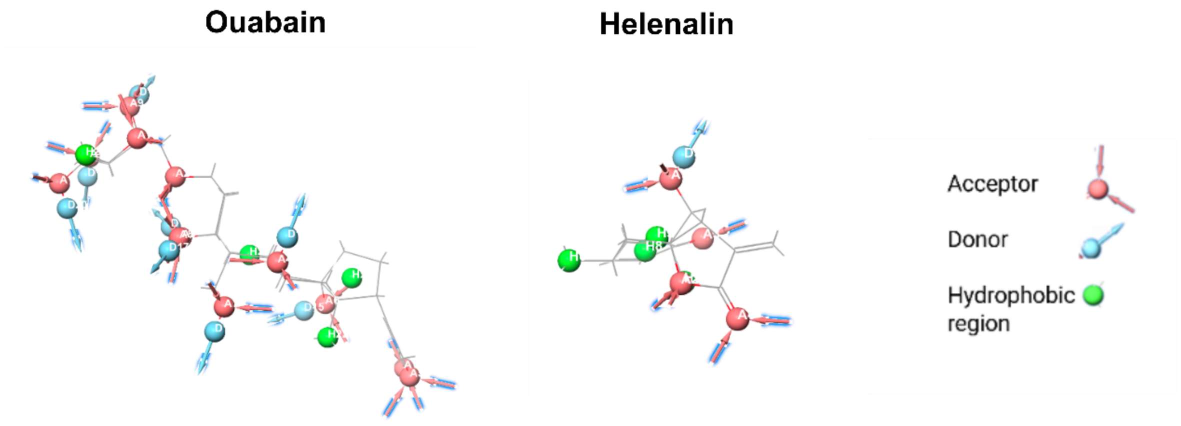

Supplementary Figure 1.
Ouabain possesses a lactone ring and hydroxyl groups, with hydrogen donors (in red, Figure S1), such as -OH groups, contributing to hydrogen bonding. Ouabain's hydrogen acceptors (in blue, Figure S1) include the oxygen atoms of the lactone ring, and it has a hydrophobic region (in green, Figure S1) around its steroidal backbone. Helenalin features an electrophilic α-methylene-γ-lactone moiety that acts as a hydrogen acceptor (in blue, Figure S1), along with hydroxyl groups as hydrogen donors (in red), and hydrophobic regions in its terpenoid framework (in green, Figure S1).

.

A

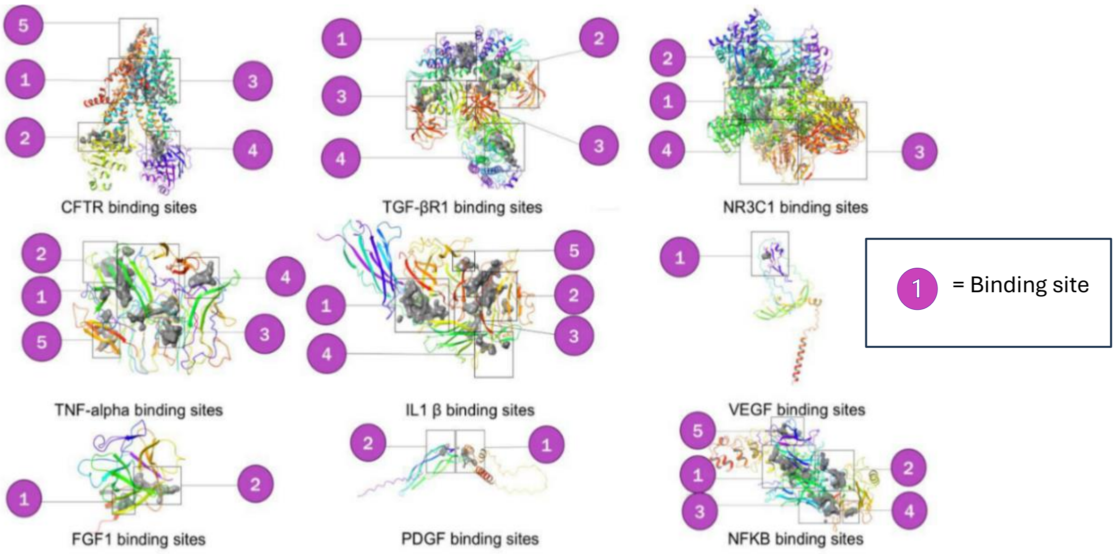

B

Protein + binding site	Pirfenidone (kcal/mol)	Ivacaftor (kcal/mol	Nintedanib (kcal/mol	Dexa-methasone (kcal/mol	Ouabain (kcal/mol	Helenalin (kcal/mol	Gentamicin C (kcal/mol)
CFTR S1		-4.088			-8.990	-4.319	-5.376
CFTR S2		-3.628			-6.236	-4.446	-5.374
CFTR S3		-5.183			-9.140	-6.027	-6.265
CFTR S4		-3.107			-7.242	-5.448	-6.351
CFTR S5		-2.628			-2.692	-4.530	-3.450
FGF1 S1						-5.111	-4.420
FGF1 S2			-1.013		-4.554		-3.845
PDGF S1			-3.848		-7.276	-3.839	-5.281
PDGF S2			-2.581		-6.747	-4.409	-5.739
VEGF S1			-0.917		-3.446	-1.194	-4.019
IL1B S1	-3.818				-8.078	-3.573	-6.489
IL1B S2	-3.888					-3.284	-5.591
IL1B S3	-2.993				-4.974	-2.180	-5.759
IL1B S4	-1.878				-6.139	-2.709	-4.787
IL1B S5	-2.833				-5.911	-4.934	-4.966
TNF S1	-3.526					-4.043	-7.066
TNF S2	-3.268				-6.169	-3.013	-5.933
TNF S3	-2.947				-7.351	-3.746	-5.885
TNF S4	-1.497				-3.606		-4.020
TNF S5	-0.978				-5.545	-1.307	-3.693
NR3C1 S1				-5.145	-7.237	-5.326	-5.892
NR3C1 S2				-3.270	-7.943	-2.704	-5.718
NR3C1 S3				-3.931	-7.531	-4.149	-5.883
NR3C1 S4				-3.078		-2.443	-5.490
NFKB S1				-2.942	-7.308	-3.955	-4.915
NFKB S2				-4.192	-5.559	-4.143	-5.044
NFKB S3				-3.765	-8.545	-2.855	-5.223
NFKB S4				-4.598	-8.469	-3.663	-5.622
NFKB S5				-4.267	-5.808	-2.858	-3.639
TGFB S1	-4.193				-9.143	-3.903	-8.801
TGFB S2	-3.930				-8.800	-4.871	-8.566
TGFB S3	-4.357				-7.881	-3.646	-7.764
TGFB S4	-4.597				-7.007	-3.986	-7.117
TGFB S5	-1.872				-8.246	-2.673	-4.223
FGFR1			-5.323		-5.280	-5.869	
FGFR2			-4.209		- 3.987	-4.329	
FGFR3			- 3.639		-1.880	- 3.923	
Na/K-ATPase					-2.283		
NFKBp65						-4.143	
PDGFR A			-5.259		-3.250	- 3.959	
PDGFR b			- 4.108		-1.940	- 4.546	
VGFR1			- 5.229		-6.123	- 3.715	
VGFR2			0.054		- 3.849	- 4.505	
VGFR3			-5.292		-4.414	-5.409	

Supplementary Figure 2. Optimal binding sites of key fibrotic proteins and docking analysis for selected drugs. A) Optimal binding sites for CFTR, TGF-βR1, NR3C1, TNF-α, IL1β, PDGF, VEGF, and NFKB. B) Full results of the binding analysis for pirfenidone, ivacaftor, nintedanib, dexamethasone, ouabain, helenalin and gentamicin C

A

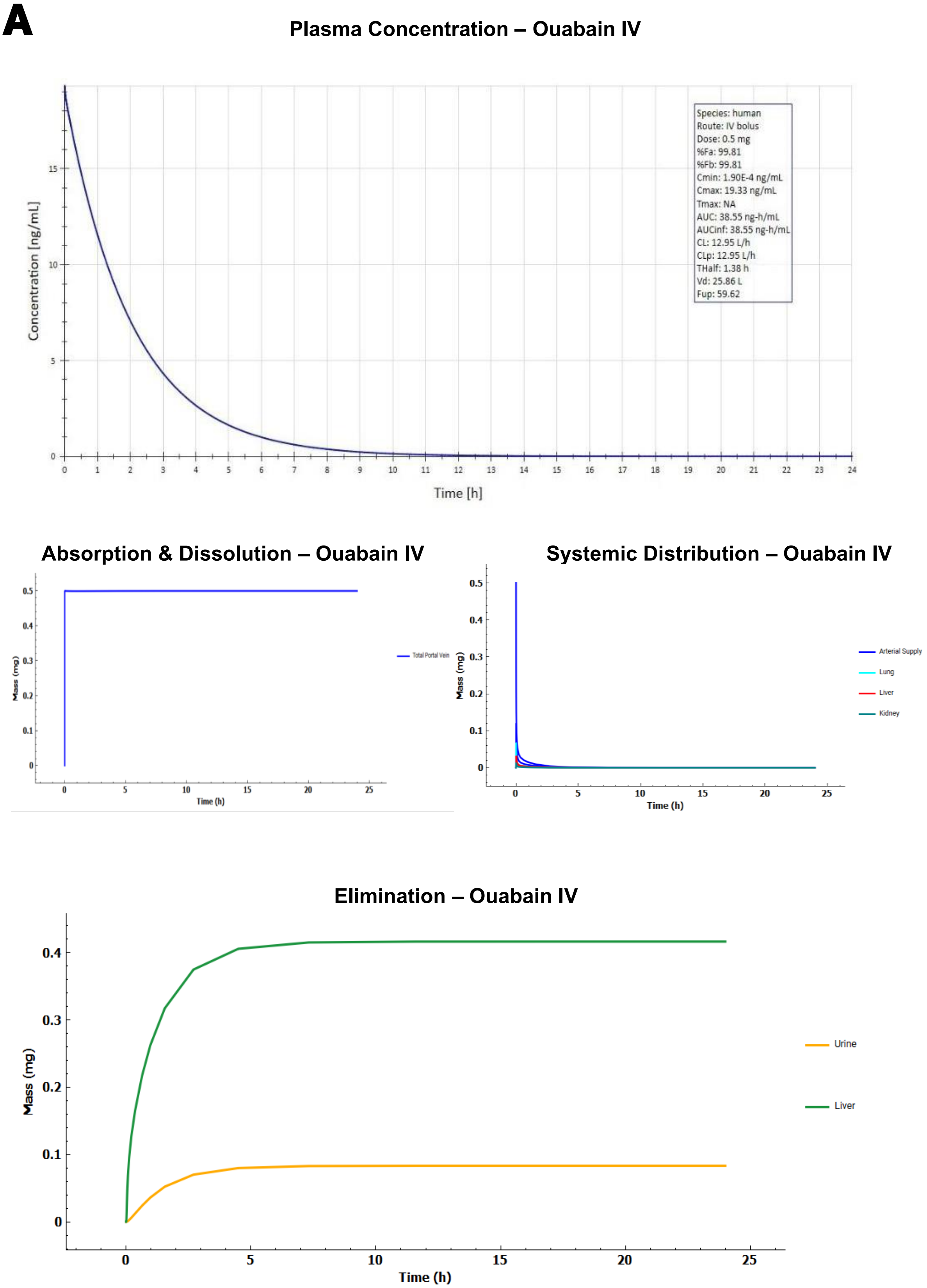

Supplementary Figure 3. The full results of PBPK models and the used parameters. A) Ouabain, IV B) Helenalin, IV C) Ouabain, oral D) Helenalin, oral E) Ouabain, inhalation F) Helenalin, inhalation

B

Plasma Concentration – Helenalin IV

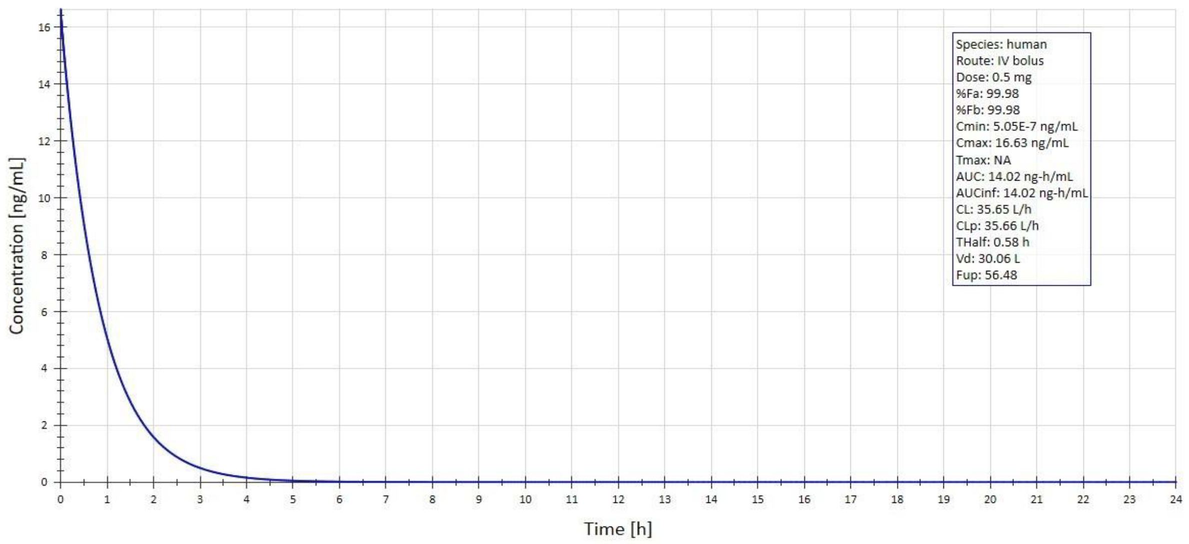

Absorption and Dissolution – Helenalin IV

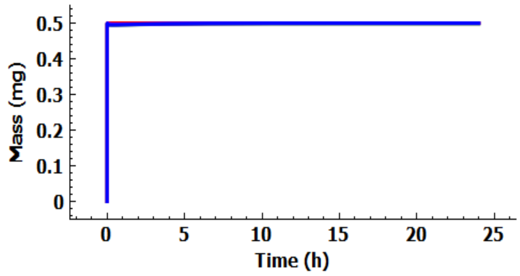

Systemic Distribution – Helenalin IV

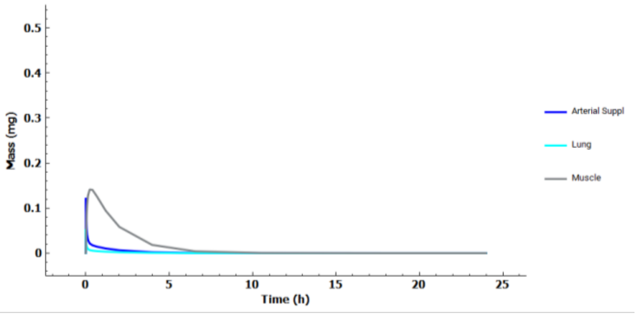

Elimination – Helenalin IV

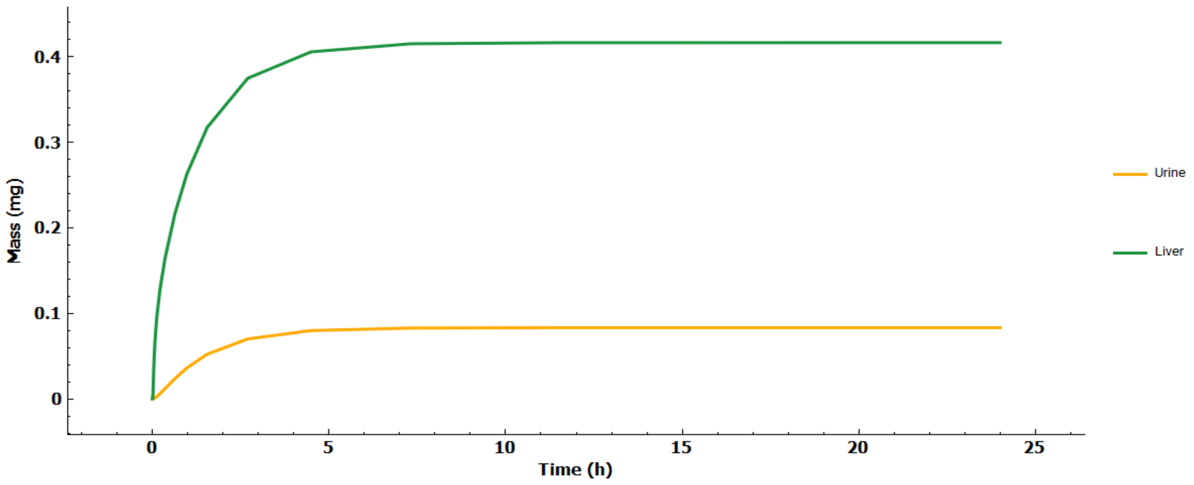

Supplementary Figure 3. The full results of PBPK models and the used parameters. A) Ouabain, IV B) Helenalin, IV C) Ouabain, oral D) Helenalin, oral E) Ouabain, inhalation F) Helenalin, inhalation

C

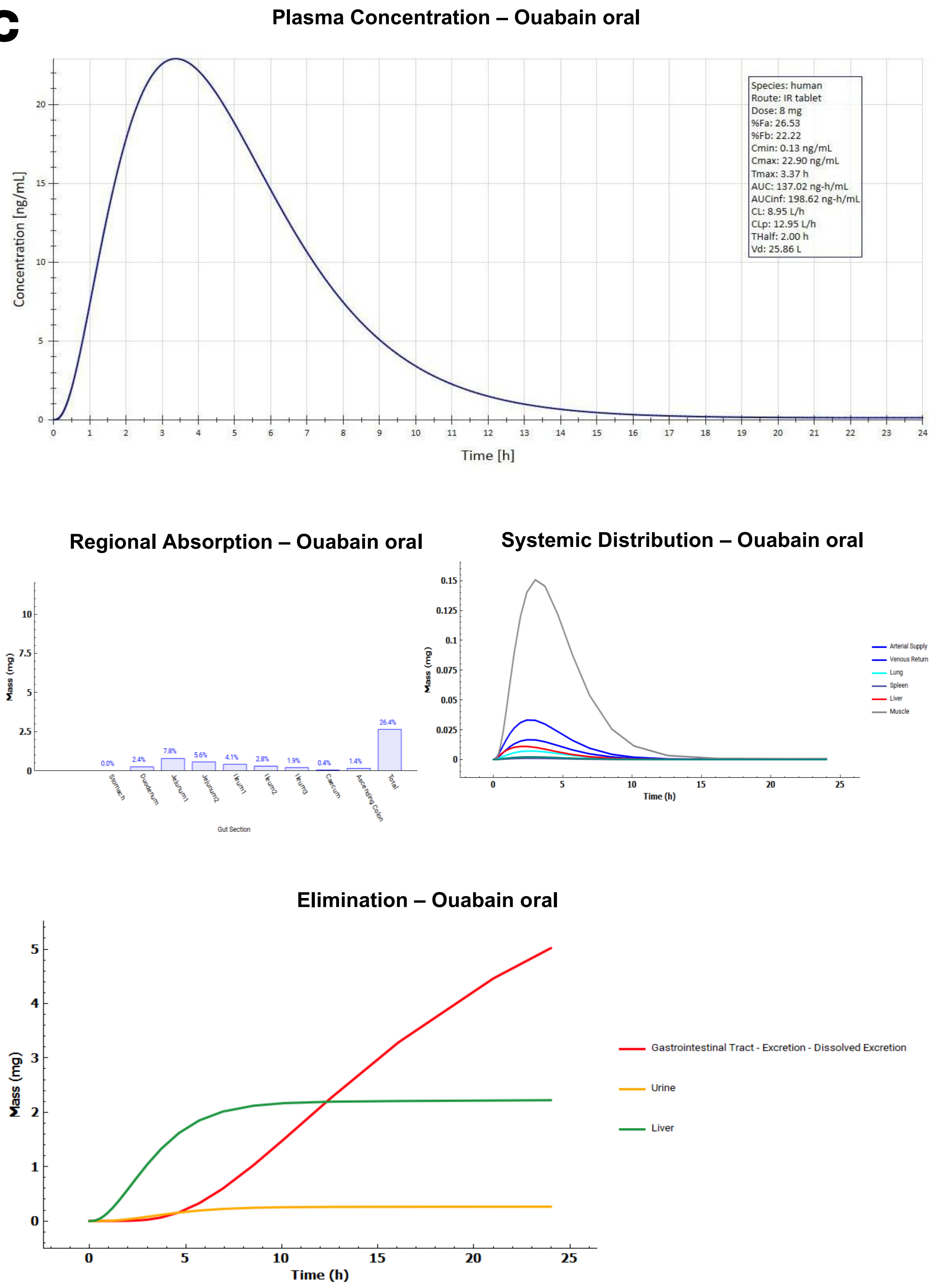

Supplementary Figure 3. The full results of PBPK models and the used parameters. A) Ouabain, IV B) Helenalin, IV C) Ouabain, oral D) Helenalin, oral E) Ouabain, inhalation F) Helenalin, inhalation

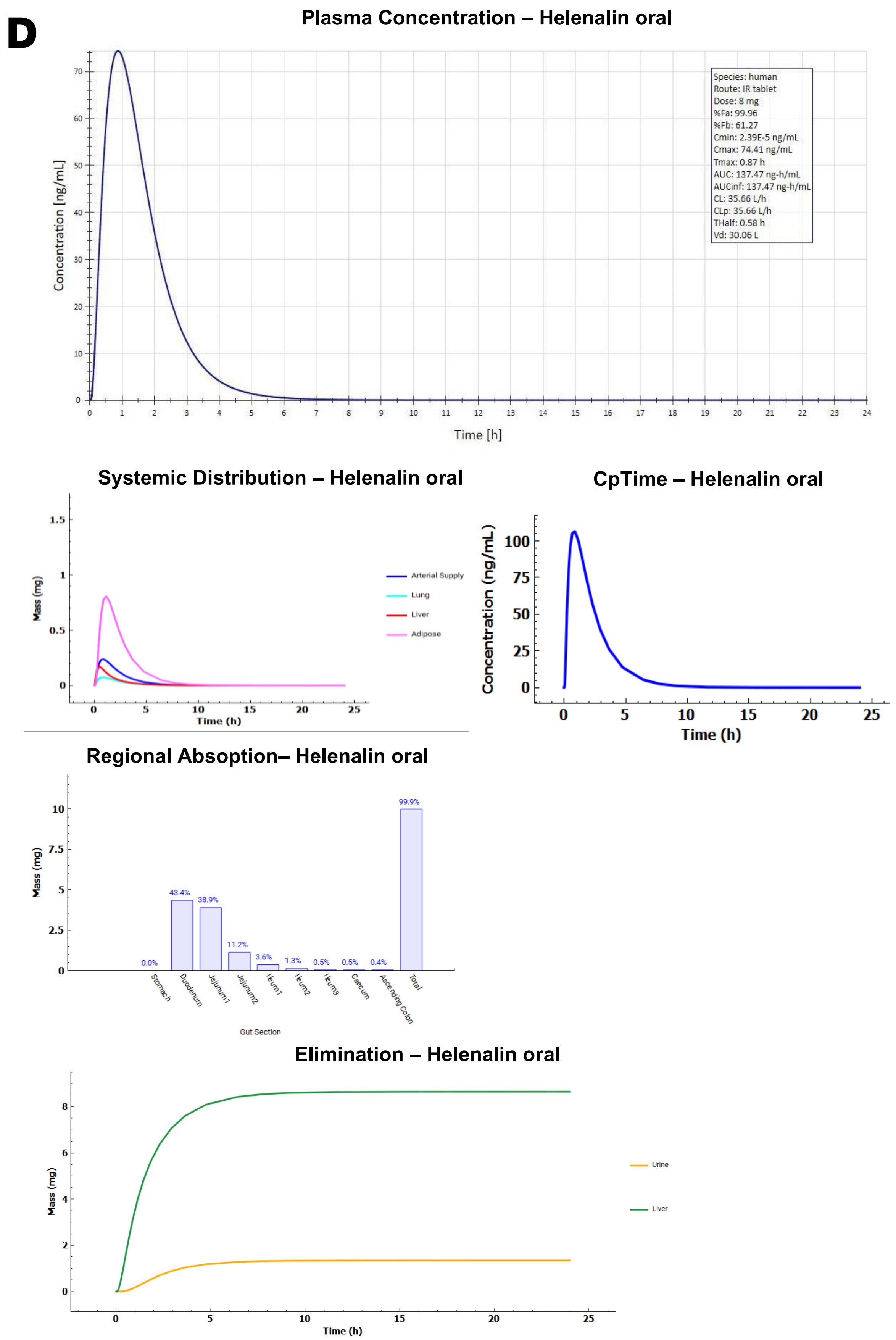

Supplementary Figure 3. The full results of PBPK models and the used parameters. A) Ouabain, IV B) Helenalin, IV C) Ouabain, oral D) Helenalin, oral E) Ouabain, inhalation F) Helenalin, inhalation

E

Simulation Output – Ouabain inhalation

Category	Variable	Value
PK Output	Fa(%)	54.304
PK Output	FDp(%)	0.246
PK Output	F(%)	54.244
PK Output	Cmax (μg/mL)	0.277
PK Output	AUC_0–t (μg·h/mL)	3.801
PK Output	AUC_0–t (μg·h/mL)	3.801
PK Output	Cmax Liver (μg/mL)	1.711
Compartmental Absorption	Pulmonary (%)	47.6
Lung PBPK Compartments	Extra-thoracic (%)	18.31
Lung PBPK Compartments	Thoracic (%)	8.44
Lung PBPK Compartments	Bronchiolar (%)	13.59
Lung PBPK Compartments	Alveolar-Interstitial (%)	34.93

Plasma Concentration– Ouabain inhalation

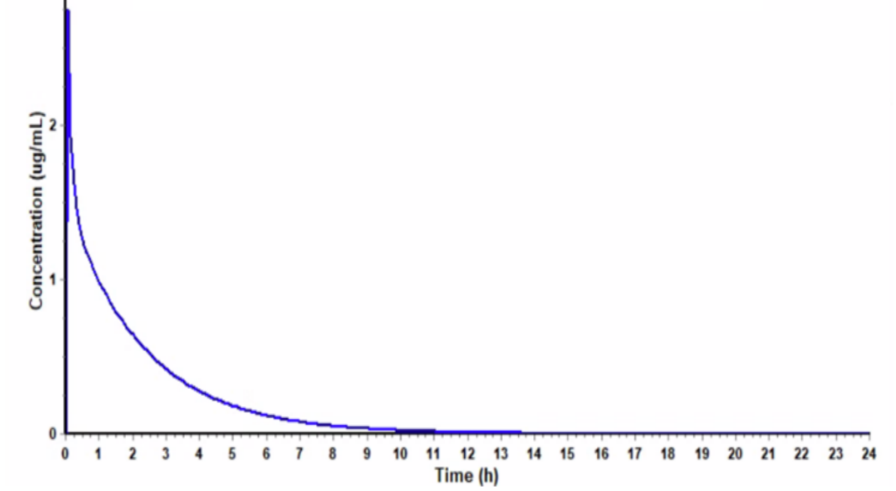

Compartmental Absorption – Ouabain inhalation

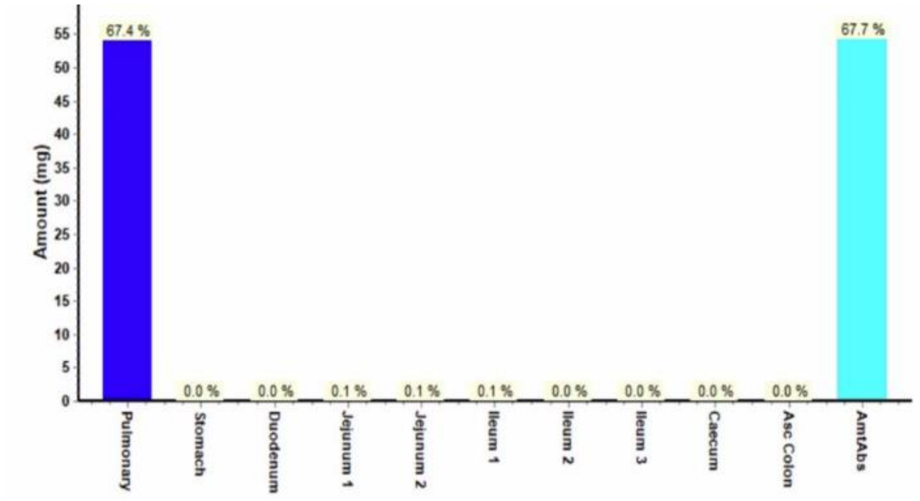

Supplementary Figure 3. The full results of PBPK models and the used parameters. A) Ouabain, IV B) Helenalin, IV C) Ouabain, oral D) Helenalin, oral E) Ouabain, inhalation F) Helenalin, inhalation

F

Simulation Output – Helenalin inhalation

Category	Variable	Value
PK Output	Fa(%)	58.23
PK Output	FDp(%)	1.37
PK Output	F(%)	57.93
PK Output	Cmax (μg/mL)	2.054
PK Output	AUC_0–t (μg·h/mL)	2.2218
PK Output	AUC_0–t (μg·h/mL)	2.2218
PK Output	Cmax Liver (μg/mL)	1.220
Compartmental Absorption	Pulmonary (%)	68.5
Lung PBPK Compartments	Extra-thoracic (%)	17.68
Lung PBPK Compartments	Thoracic (%)	7.02
Lung PBPK Compartments	Bronchiolar (%)	15.06
Lung PBPK Compartments	Alveolar-Interstitial (%)	32.49

Plasma Concentration– Helenalin inhalation

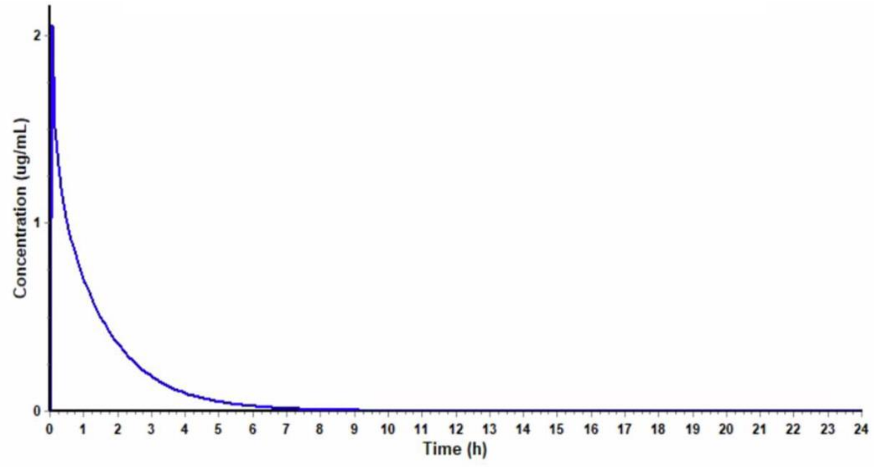

Compartmental Absorption – Helenalin inhalation

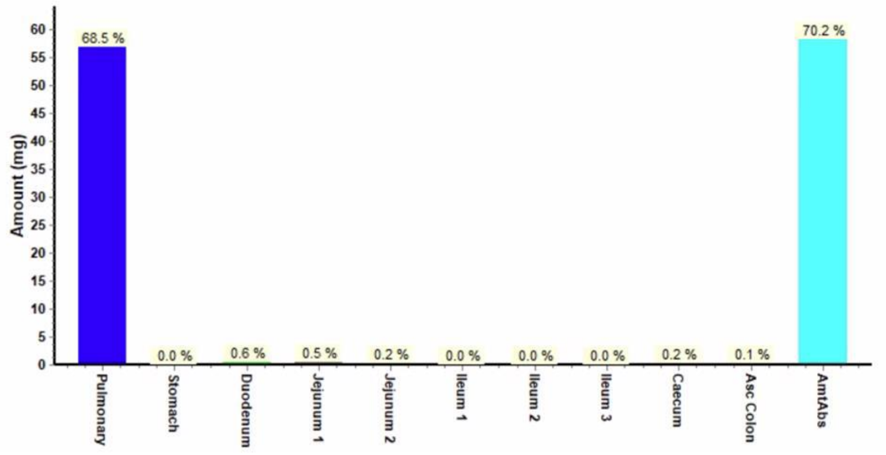

Supplementary Figure 3. The full results of PBPK models and the used parameters. A) Ouabain, IV B) Helenalin, IV C) Ouabain, oral D) Helenalin, oral E) Ouabain, inhalation F) Helenalin, inhalation

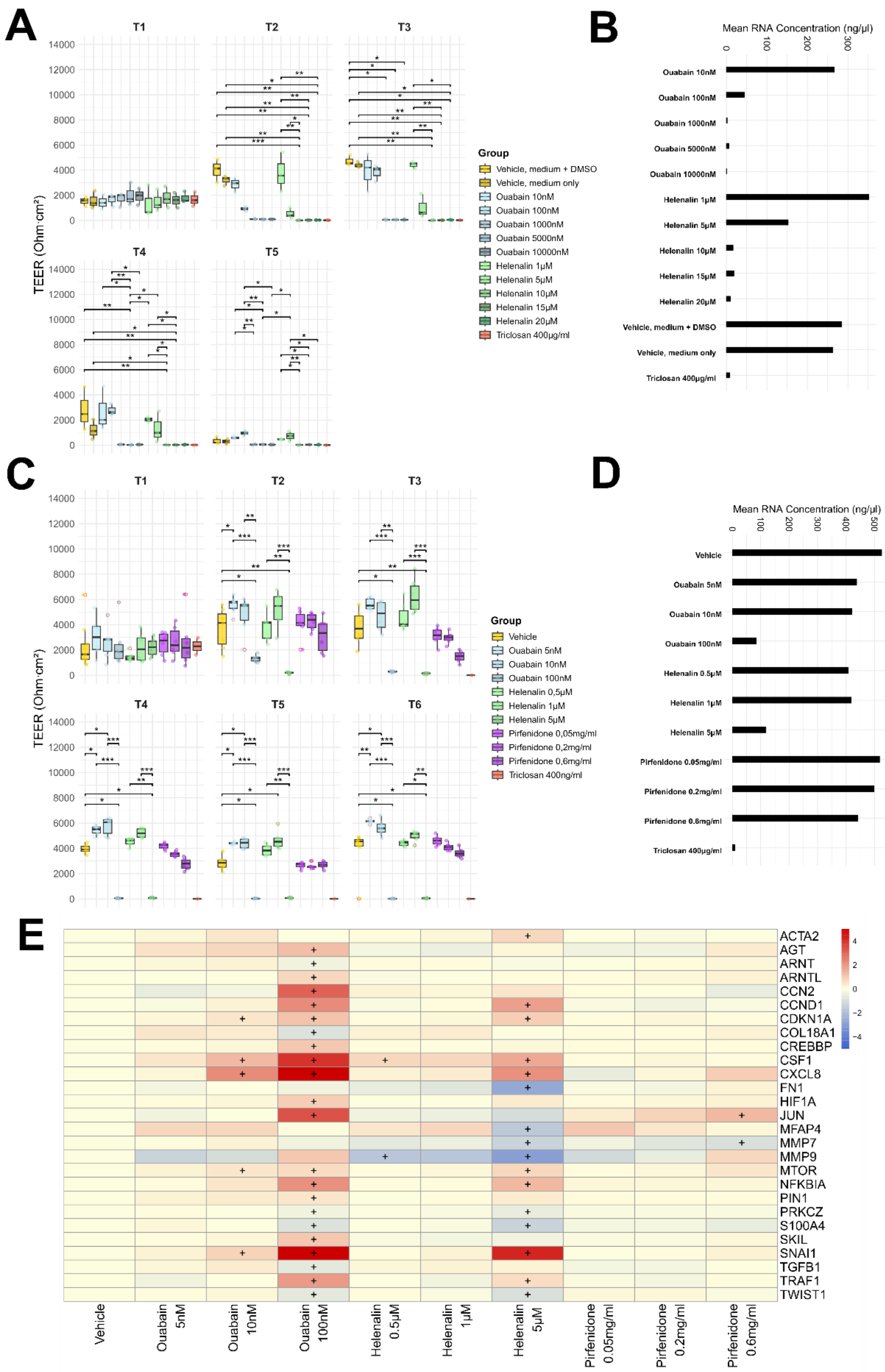

Supplementary Figure 4. Results of the TEER, RNA yield and RNA expression analysis of helenalin, ouabain and pirfenidone dose-finding studies. **A- B)** TEER and RNA yield. Exposures were given after TEER measurements on days 1 and 3 (T1 and T3). RNA was collected at day 5 after starting exposures (T5). **C-D)** TEER and RNA yield in the second test where helenalin, ouabain and pirfenidone were administered daily **E)** PCR results from the second test (only significant results are shown). For A and C the unadjusted p-values are presented for relevant comparisons. *p<0.05, **p<0.01, ***p<0.001

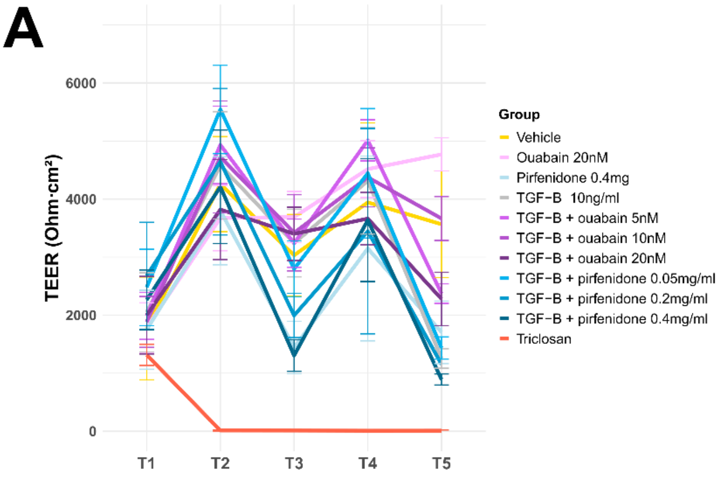

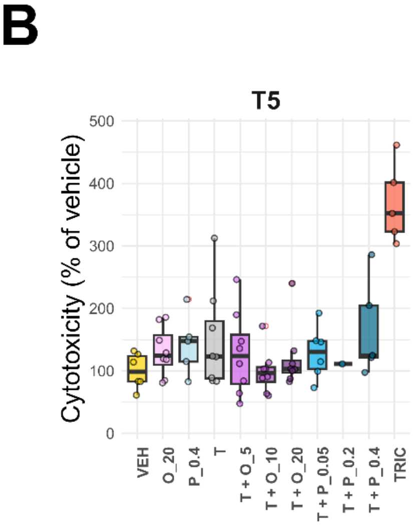

Comparison	Timepoint	P.adj
TGF-B 10ng/ml + ouabain 5nM - TGF-B 10ng/ml + ouabain 20nM	T4	0,036506
TGF-B 10ng/ml + pirfenidone 0,4mg/ml - Vehicle	T5	0,012130
Ouabain 20nM - Pirfenidone 0,4mg/ml	T5	0,039253
TGF-B 10ng/ml + ouabain 10nM - TGF-B 10ng/ml	T5	0,014926
Ouabain 20nM - TGF-B 10ng/ml	T5	0,000180

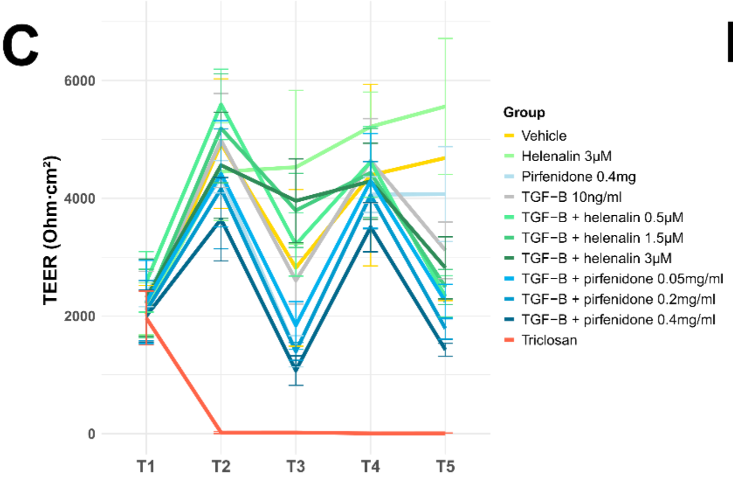

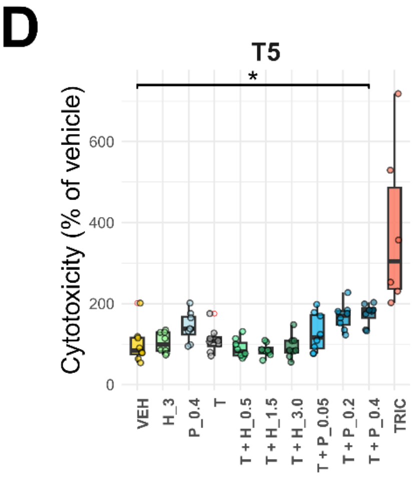

Comparison	Timepoint	P.adj
Helenalin 3µM-Vehicle	T3	0,000241
Pirfenidone 0,4mg/ml-Vehicle	T3	0,004846
TGF-B 10ng/ml + pirfenidone 0,2mg/ml-Vehicle	T3	0,004768
TGF-B 10ng/ml + pirfenidone 0,4mg/ml-Vehicle	T3	0,000164
Pirfenidone 0,4mg/ml-Helenalin 3µM	T3	0,000000
TGF-B 10ng/ml-Helenalin 3µM	T3	0,000022
TGF-B 10ng/ml + helenalin 0,5µM-Helenalin 3µM	T3	0,012739
TGF-B 10ng/ml-Pirfenidone 0,4mg/ml	T3	0,033020
TGF-B 10ng/ml + helenalin 1,5µM-TGF-B 10ng/ml	T3	0,036980
TGF-B 10ng/ml + helenalin 3µM-TGF-B 10ng/ml	T3	0,008905
TGF-B 10ng/ml + pirfenidone 0,2mg/ml-TGF-B 10ng/ml	T3	0,032567
TGF-B 10ng/ml + pirfenidone 0,4mg/ml-TGF-B 10ng/ml	T3	0,001596
TGF-B 10ng/ml + pirfenidone 0,4mg/ml - Vehicle	T5	0,003956
TGF-B 10ng/ml + pirfenidone 0,2mg/ml - Pirfenidone 0,4mg/ml	T5	0,016242
TGF-B 10ng/ml + pirfenidone 0,4mg/ml - Pirfenidone 0,4mg/ml	T5	0,000857
TGF-B 10ng/ml + pirfenidone 0,4mg/ml - TGF-B 10ng/ml	T5	0,019312

Supplementary Figure 5. Full results of the TEER, viability and cytotoxicity measurements and statistics related to Figure 6. **A, C)** TEER, and **B,D**) cytotoxicity measurements

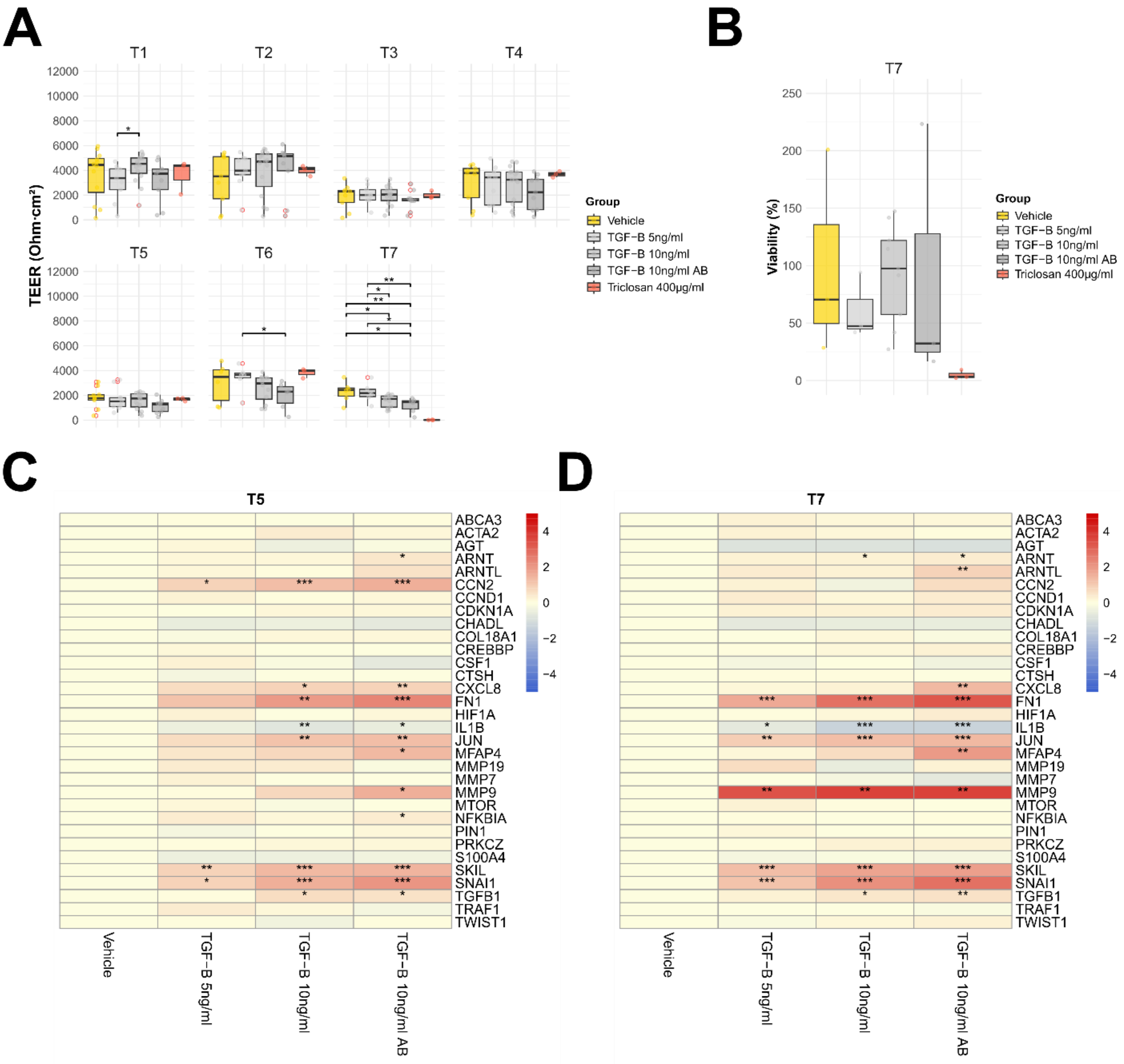

Supplementary Figure 6. Results of the dose-range-optimization study of TGF-B exposure. A) The effect of daily TGF-B exposures on huArlo barrier. Unadjusted statistics are shown between all other groups except triclosan. B) The effect of repeated TGF-B exposure on huArlo cell viability C-D) The effect of daily TGF-B exposures on huArlo RNA expression after four days (T5) and six days (T7). *$p<0.05$, **$p<0.01$, ***$p<0.001$

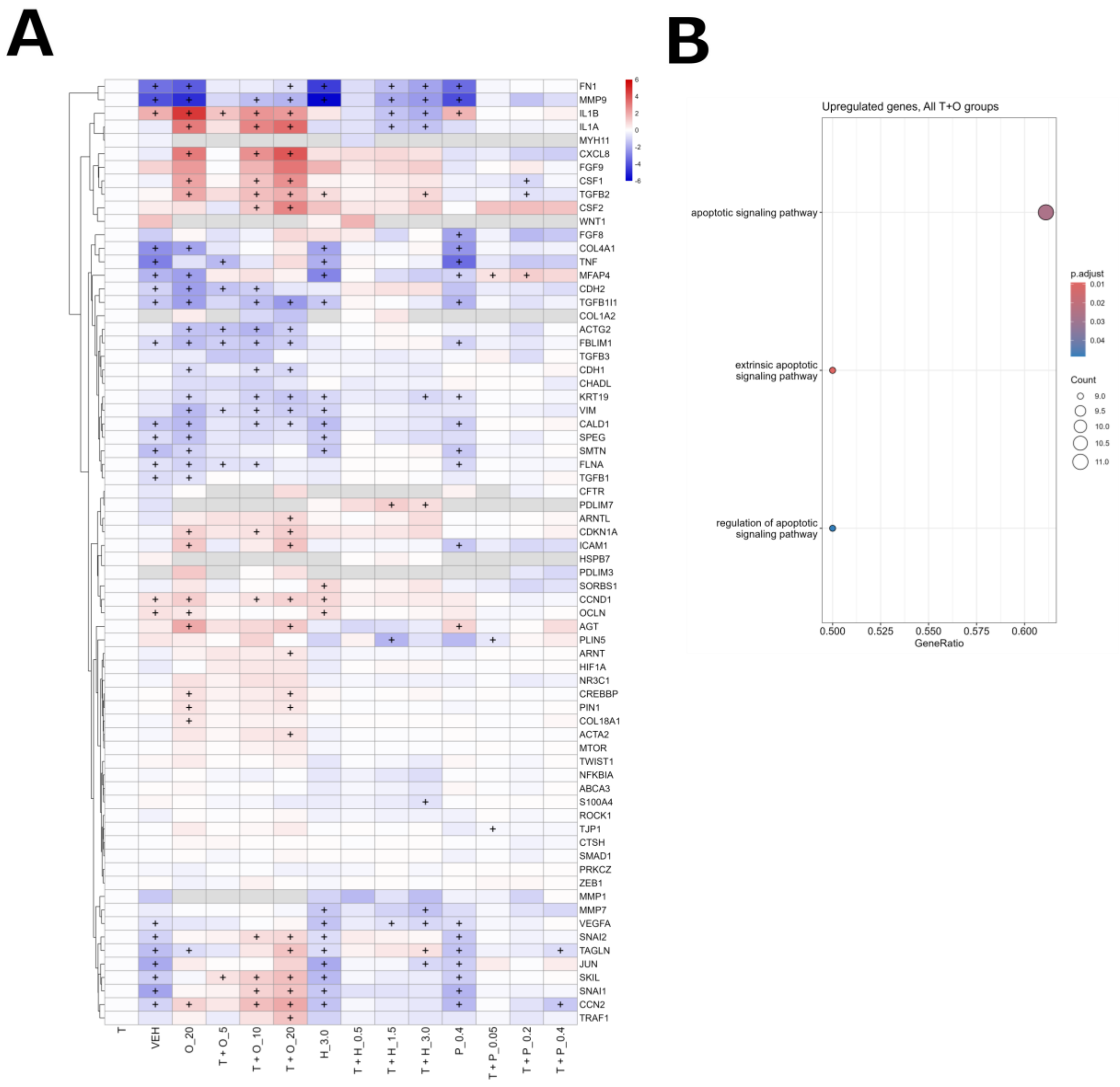

Supplementary Figure 7. A) Full analysis results related to Figure 7. Heatmap presents all analysed log2Fold values from the data. Blue color indicates downregulation and red upregulation of a gene. Differentially expressed genes (p-adj <0.05 (FDR)) marked with "+". Grey = no data. **B)** Gene ontology analysis of differentially expressed genes (p-adj <0.05 (FDR) from the TGF-β 10ng/ml and ouabain groups. Other groups did not result in enriched pathways.
